# What treatment and services are effective for people who are homeless and use drugs? A systematic ‘review of reviews’

**DOI:** 10.1371/journal.pone.0254729

**Published:** 2021-07-14

**Authors:** Joanna Astrid Miler, Hannah Carver, Wendy Masterton, Tessa Parkes, Michelle Maden, Lisa Jones, Harry Sumnall

**Affiliations:** 1 Salvation Army Centre for Addiction Services and Research, Faculty of Social Sciences, University of Stirling, Stirling, Scotland; 2 Faculty of Social Sciences, University of Stirling, Stirling, Scotland; 3 Institute of Population Health Sciences, University of Liverpool, Liverpool, England; 4 Public Health Institute, Liverpool John Moores University, Liverpool, England; University of Birmingham, UNITED KINGDOM

## Abstract

**Background:**

People who experience homelessness and those vulnerably housed experience disproportionately high rates of drug use and associated harms, yet barriers to services and support are common. We undertook a systematic ‘review of reviews’ to investigate the effects of interventions for this population on substance use, housing, and related outcomes, as well as on treatment engagement, retention and successful completion.

**Methods and findings:**

We searched ten electronic databases from inception to October 2020 for reviews and syntheses, conducted a grey literature search, and hand searched reference lists of included studies. We selected reviews that synthesised evidence on any type of treatment or intervention that reported substance use outcomes for people who reported being homeless. We appraised the quality of included reviews using the Joanna Briggs Institute Critical Appraisal Checklist for Systematic Reviews and Research Syntheses and the Scale for the Assessment of Narrative Review Articles. Our search identified 843 citations, and 25 reviews met the inclusion criteria. Regarding substance use outcomes, there was evidence that harm reduction approaches lead to decreases in drug-related risk behaviour and fatal overdoses, and reduce mortality, morbidity, and substance use. Case management interventions were significantly better than treatment as usual in reducing substance use among people who are homeless. The evidence indicates that Housing First does not lead to significant changes in substance use. Evidence regarding housing and other outcomes is mixed.

**Conclusions:**

People who are homeless and use drugs experience many barriers to accessing healthcare and treatment. Evidence regarding interventions designed specifically for this population is limited, but harm reduction and case management approaches can lead to improvements in substance use outcomes, whilst some housing interventions improve housing outcomes and may provide more stability. More research is needed regarding optimal treatment length as well as qualitative insights from people experiencing or at risk of homelessness.

## Introduction

Homelessness encompasses a range of housing situations including both sheltered (e.g. temporary accommodation) and unsheltered settings (e.g. the streets), but lacks a standardised definition [1, 2]. FEANTSA have previously developed a typology seeking to define homelessness in an operational way [3]. Through this, homelessness can be defined based on four categories: rooflessness; houselessness; insecure housing; and inadequate housing [3]. The Canadian Observatory on Homelessness (COH) have also developed a typology in an attempt to improve understanding of the term [4]. Similar to FEANTSA, COH define homelessness as encompassing a range of living situations including: people living unsheltered; people who are in emergency shelters; people who are in temporary accommodation; and those at risk of homelessness and whose housing situations are precarious [4]. In the UK and Irish policy context, the definition of homelessness is also typically expanded to include people ‘at risk’ of homelessness. Recent estimates suggest that 307,000 people in the UK [5], 567,715 in the USA [6], and 235,000 in Canada [7], experience homelessness in a year, with the numbers increasing [8]. Due to variation in the definition of homelessness the true magnitude of the problem may be higher still. The route into homelessness is complex and is generally a result of many contributing factors. Systemic or societal barriers are key drivers, for example lack of affordable housing, access to resources, or discrimination [4]. Poverty is also an important factor [9], with COH reporting that homelessness is directly linked to the inequalities in financial support for people who are often in crisis situations [4]. Other individual cirumstances can increase a person’s risk of homelessness, including childhood trauma, mental health problems, substance use, and previous imprisonment [10].

People who are homeless, and those who are vulnerably housed (defined as experiencing prior homelessness or having frequent housing transitions [11]), experience disproportionately high rates of substance use [12–14], as well as poorer physical [12, 14] and mental health [15–17] than the general population. People who are homeless also have a higher risk of developing health problems that are relatively rare within the general population, such as those caused by blood-borne viruses (BBVs) including hepatitis and human immunodeficiency virus (HIV) [17, 18]. Moreover, the longer a person is homeless, the higher their risk of ill health and premature death [19], with mortality rates estimated to be between three to four times higher than in the general population [14, 20].

Despite higher rates of physical and mental ill health, people who are homeless attend primary care and preventive services, such as screenings and check-ups, less often than the general population [21]. Barriers to accessing appropriate care can include: negative previous experiences of such care; other priorities such as shelter and food; and access barriers such as perceived prejudice and judgemental staff, poor coordination between healthcare services, cost of medication, lack of continuity of care, challenges with strict appointment times, and complex administrative processes [21, 22]. These barriers can lead to delayed or no treatment which, in turn, can increase the risks of more serious health problems [23]. Indeed, globally, the rate of hospital admissions for people who are homeless has been shown to be between two and five times higher than for the general population [24].

Individuals experiencing homelessness are also less likely to access, and more likely to disengage from, substance use treatment [25]. Individuals may use substances as a way to cope with the trauma of homelessness, stress, and adversity [26–28]. Previous trauma experienced both in childhood and adulthood, as well as vicarious trauma and posttraumatic stress disorder, can also influence substance use [29]. Despite the considerable unmet care needs of this population, people who experience both homelessness and problem substance use (defined as ‘the use of drugs and/or alcohol in a way that had a negative effect on their lives’) often face overlapping barriers to accessing care. These include stigma related to care itself [30], as well as sub-optimal treatment lengths and judgemental staff [31]. Moreover abstinence-based Treatment First [TF] housing services can be inaccessible to many of those in need of housing, creating more difficulties [32, 33]. Together, these barriers can contribute to mistrust of health services, maintenance of low levels of access and adherence to care, and an increase in people’s perceived loss of control and lack of mastery over their lives [34–36].

Existing treatment options for problem substance are diverse, and can be placed on a continuum ranging from harm reduction to abstinence-based approaches. Harm reduction approaches include pragmatic interventions, policies, and programmes, but do not require a person to stop using drugs as a condition of support [37]. Research evidence and policy guidance supports provision of harm reduction and abstinence orientated actions depending upon target population need [22, 31, 38]. Evidence regarding how treatment for problem substance use is best delivered to those experiencing homelessness is limited, although engaging, flexible services have been shown to be important [39, 40]. For those who have successfully accessed treatment, challenges associated with continued engagement with treatment and recovery as a result of being homeless often remain [31].

Several systematic reviews and primary research studies have examined the effectiveness of various specific interventions (such as case management or Housing First (HF) approaches) for people who are homeless, and for people with problem substance use. However, evidence that pools and synthesises the available data is lacking. Moreover, evidence pertaining specifically to people who experience both homelessness and problem substance use is limited. This ‘systematic review of reviews’ aimed to address this gap by synthesising all available evidence on the effectiveness of treatments and interventions for this specific population. The review includes housing interventions, peer support interventions, and harm reduction approaches, among others. This review evaluates the effects of these interventions on those who use services (referred to as ‘clients’ throughout the review), regarding substance use, housing, and ‘other’ outcomes, as well as on treatment entry, engagement, retention and successful completion. We also identified components of good practice.

## Methods

### Study design

This systematic review of reviews provides a synthesis of international evidence regarding interventions in primary care, mental health, and drug treatment settings, for people who are homeless who use drugs. Given the large body of existing evidence available on the topic, a systematic review of reviews was considered to be the most appropriate approach. The review methodology proceeded in accordance with guidelines from the Joanna Briggs Institute [41], and was reported according to the Preferred Reporting Items of Systematic Reviews and Meta-Analyses (PRISMA) guidelines [42] (S1 PRISMA checklist). No protocol was registered with an open-access registry (e.g. PROSPERO) prior to publication.

This review was undertaken as part of a larger piece of research commissioned by the Health Research Board, Ireland, and undertaken by the same authors in 2019–2020 [43]. The larger study combined an analysis of current drug trends and provision of services in Ireland (with contextual mapping) with the systematic review. This current review provides an updated search and new data. The main outcomes of this review focused on: i) substance use; ii) housing; and iii) ‘other’ outcomes. We also extracted and synthesised, where possible, information regarding treatment entry/engagement and retention (engaging the population of interest to enter treatment/engage with a service), and successful completion of treatment (attrition rates throughout treatment duration).

### Search strategy and selection criteria

The PICOS framework (population, interventions, comparators, outcomes, and study design) [44] was used to formulate the inclusion/exclusion criteria (see Table 1) and identify appropriate literature search terms.

**Table 1:** Inclusion/exclusion criteria.

Inclusion	Exclusion
**P**opulations	
People experiencing homelessness and drug use (including poly-substance use–i.e. concurrent use of various substances)	People who are not deemed homeless; alcohol or tobacco use only
Range of drugs used both problematically and/or recreationally, including PIEDs	
	Non-drug use
Adults (over 18 years, with no upper age limit)	Under 18s
**I**nterventions	
Problem drug use treatment (including poly-substance use)	Non-drug related interventions and treatment
Harm reduction approaches	
Interventions in primary care for drug use	
Interventions in mental health settings for drug use	Alcohol or tobacco only interventions
Residential rehabilitation	
Detoxification	
**C**omparators	
Any	
**O**utcomes	
Reduced drug consumption	Non-drug related outcomes
Reduced overdoses (fatal and non-fatal)	
Reduced drug related harm	Alcohol only related outcomes
Improved quality of life	
Improved health outcomes	
Improved housing outcomes	
**S**tudy design	
Review (including systematic review, meta-analysis, evidence synthesis, realist review, mixed methods review, qualitative synthesis, meta-epidemiology, integrative review, umbrella review, critical interpretative synthesis)	Primary research
	Literature search

An information specialist (MM) led the development and application of the search strategies, supported by all members of the research team. The searches were conducted across 10 electronic databases (see Table 2). All searches were run on 30 December 2019, with an updated search conducted on 3 October 2020. We also searched a range of organisational websites from December 2019 to January 2020 to ensure that any relevant reviews situated in the grey literature were identified (S1 Table). Full search strategies can be found in S1 Data. Reference details identified through the literature search were collated and managed using EndNote. Reference lists of included articles were screened for additional reviews. No date or language restrictions were included in order to minimise bias and ensure that all relevant reviews could be captured. Two reviews written in languages other than English (Canadian French and Spanish) were included, translated via Google Translate and deemed of acceptable quality by the research team for the purposes of data extraction.

**Table 2:** Databases searched.

**Database**
MEDLINE (Ovid)
CINAHL (EBSCOhost)
Embase (Ovid)
PsycINFO (Ovid)
PROSPERO
Epistemonikos
Cochrane Database of Systematic Reviews
Joanna Briggs Institute Database of Systematic Reviews
Heath Technology Assessments (via National Institute for Health Research Journals)
The Campbell Collaboration

One reviewer (JM) screened all titles and abstracts, alongside the full-text of articles that were considered relevant. A second reviewer (WM) independently assessed 20% of all titles and abstracts to ensure inter-rater reliability, as deemed to be good practice in rapid systematic review methodology [45]. The relevance of each article was assessed according to the criteria set out in Table 1. Any discrepancies were resolved by consensus or, if necessary, by consulting a third reviewer (HC). As a second reliability check TP, HC, WM, and JM discussed all identified relevant papers in consultation with HS. By consensus, it was agreed that only reviews where at least 40% of all included papers were relevant to substance use and homelessness were to be included, to ensure that the review maintained a firm focus on both topics. Adopting a minimum percentage in this context has also been used in other systematic reviews [46]. Reviews of both quantitative and qualitative studies were included, as were non-systematic reviews. Papers reporting pooled data or meta-analyses without an accompanying systematic review were rejected.

### Quality assessment

Reviews were not excluded based on quality appraisal scores but evidence quality was noted in accordance with the recommendations proposed by the Centre for Reviews and Dissemination [47]. Two reviewers (JM and HC) independently assessed the quality of the included systematic reviews using the JBI Critical Appraisal Checklist for Systematic Reviews and Research Syntheses [41] (S2 Data); and the quality of the non-systematic reviews using the Scale for the Assessment of Narrative Review Articles (SANRA) [48] (S3 Data). Any disagreement in scores was resolved through consensus and, if necessary, by a third reviewer (WM). Overall, the quality of the included systematic reviews was moderate, with three achieving the highest possible score of 11, and six receiving a score of six or lower. The included non-systematic reviews were apparised to be of moderate to high quality. Quality appraisal allowed for the study strengths and weaknesses to be considered but papers were not excluded based on their scores. The final scores are presented in S2 Table.

### Data analysis

Data relating to study design and key characteristics, including populations, interventions, outcomes, and implications for policy and practice, were extracted by one reviewer (JM) into an Excel spreadsheet. Data from the reports identified through the grey literature search were extracted into the same spreadsheet by a second reviewer (WM). The data extraction table (S3 Table) was shared with other team members (HC, TP, HS) to check and ensure accuracy.

As this systematic review of reviews includes both quantitative and qualitative reviews regarding diverse types of interventions and outcomes, pooling of data was not possible, and a narrative synthesis was deemed the most suitable option for data analysis. One author (JM) summarised included studies in a narrative synthesis using textual description of each study included. Thematic summaries were developed based on the type of intervention in the included studies which enabled the synthesis and supported comparisons to be made between each study [49]. Although the search focused on controlled drugs, the team also extracted data on about alcohol, prescription drug and tobacco use, if these were included. One of the reviews previously identified for inclusion [50] only presented an abstract from a conference, with the full review not available/not published. Full data extraction was therefore not possible for this paper.

## Results

**Fig 1:**
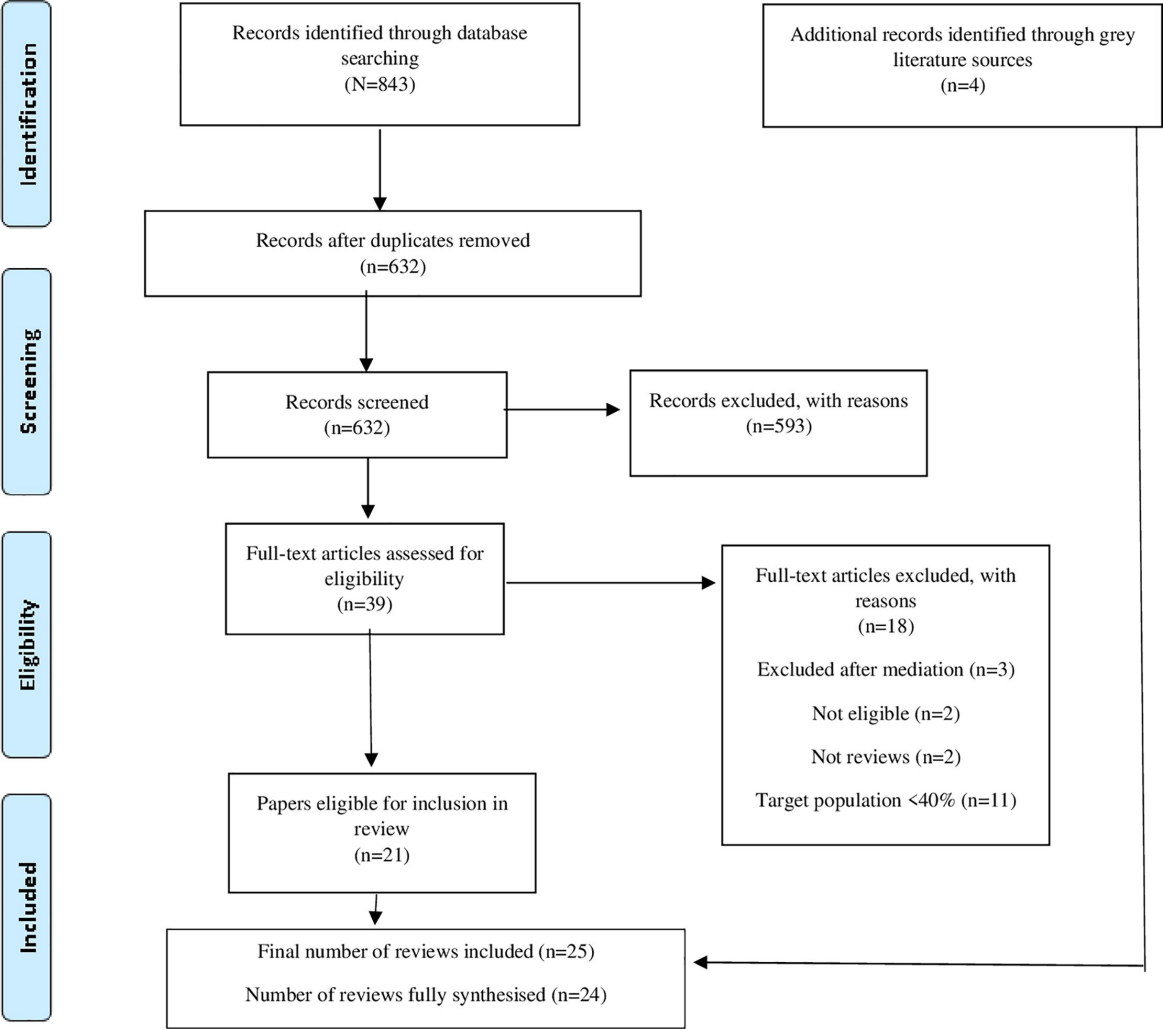
PRISMA flow diagram.

The literature searching and screening process are shown using a PRISMA flow diagram [51] (Fig 1). In total, including initial and updated searches together, 843 reviews were identified via database searches, with a further four identified in grey literature searches. Six hundred and thirty two reviews were screened against the inclusion criteria and 39 were assessed at full text, of which 18 were excluded (Fig 1). Across both searches, a total of 25 reviews were included, 24 of which were fully synthesised (full text was not available for one of the included reviews thus making its inclusion in final synthesis not possible). Twenty one reviews were published in the scientific literature, and four were grey literature reviews.

### Characteristics of the included reviews

Included reviews were published between 2004 and 2020, and consisted of: four grey literature reports [39, 52–54]; 18 systematic reviews [2, 31, 46, 50, 55–68], two of which also included a meta-analysis [2, 65]; and three non-systematic reviews [69–71]. Thirteen reviews included quantitative studies only, 11 included any study type/mixed designs, including one realist synthesis [62], two systematic review of reviews [52, 60], one ‘state of the art’ review [61], and one review was a meta-ethnography of qualitative studies [31]. The number of included studies per review ranged from four [2] to 151 [53], with five reviews not reporting how many studies were included in the final synthesis [39, 54, 68, 70, 71].

Eleven of the reviews were undertaken in the United Kingdom (UK), four in the United States of America (USA), six in Canada, three in Europe (Spain, Ireland, and a Dutch/Belgian collaboration), and one was an international collaboration (Switzerland, the UK, and Canada). Nearly all reviews (22/25) were international in focus, with two focusing on the USA and one on the UK only. The majority of primary studies were undertaken in the USA.

### Overview of the included reviews–primary focus

The included reviews were diverse in terms of their primary focus and included a range of interventions (Table 3). Two of the included reviews focused on any/all health interventions, rather than on a specific intervention type, thus they included a variety of programmes ranging from harm reduction for people who use drugs to sexual health promotion programmes.

**Table 3:** Primary focus of included reviews.

Theme	Description of intervention	Number of included papers	Reviews
Housing interventions (including Housing First (HF) initiatives)	HF focuses on providing immediate, permanent, low-barrier, non-abstinence-based supportive housing for individuals with lived experience of homelessness.	6	Baxter et al. (2019) [72]; Beaudoin (2016) [55]; Benston (2015) [56]; Chambers et al. (2017) [57]; Kertesz et al. (2009) [70]; Pleace and Quilgars (2013) [54]
Co-occurring serious mental health problems and alcohol/drug use (COSMHAD)	Residential programmes and community-based treatment. Residential programmes can integrate mental health treatment, substance use interventions, housing, and other types of support. Community-based treatment can also include integrated treatment.	4	Brunette et al. (2004) [69]; Minyard et al. (2019) [53]; O’Campo et al. (2009) [62]; Sun (2012) [71]
Case management	Case management is a strategy to support rapid rehousing, especially for those with complex needs. It provides outreach, assessment, planning, linkage, monitoring, and advocacy services. This strategy typically provides support in developing independent living skills, acute care in crisis situations, and support with medical and psychiatric treatment (de Vet et al., 2013).	4	de Vet et al. (2013) [58]; Torres Del Estal and Álvarez (2018) [64]; Penzenstadler et al. (2019) [67]; Ponka et al., (2020) [63]
Treatment for problem substance use	Treatment approaches for problem substance use are wide ranging and can be placed on a continuum, ranging from harm reduction to abstinence-based approaches.	3	Bates et al. (2017) [52]; Carver et al. (2020) [31]; Pleace (2008) [39]
Any type of healthcare/treatment/intervention	These included: adequate oral opioid maintenance therapy; tetanus and Hepatitis A, B, and C immunisations; safer injecting advice and access to NSPs; supervised consumption facilities (SCF); peer distribution of take-home naloxone (THN); assertive outreach programmes; supportive programmes for substance dependence; and sexual health promotion programmes.	2	Hwang et al. (2005) [59]; Wright and Tompkins (2006) [68]
Peer support	Peers with experience of homelessness offer support to those currently experiencing homelessness. Intentional peer support (IPS) is fostered and developed by professional organisations, formalising this process.	2	Barker and Maguire (2017) [46]; Miler et al. (2020) [61]
Harm reduction (Reviews that were specifically about harm reduction interventions for people who are homeless who use drugs)	Two important harm reduction interventions for injecting drug users are opioid substitution therapy (OST) (to reduce drug dependence and injecting frequency) and the provision of clean injecting equipment through needle and syringe programmes (NSPs); to reduce unsafe injecting, i.e. sharing used syringes). Other harm reduction interventions include THN and SCFs.	2	Turner et al. (2011) [65]; Magwood et al. (2020) [60]
Emergency department (ED) interventions	These are interventions provided/initiated at the ED, aiming to improve health and/or access to the social determinants of health. These include case management, HF, substance use interventions, and ED-based resource desks and ED compassionate care.	1	Formosa et al. (2019) [50]
Sexual health promotion	This included programmes combining HIV education; alcohol and drug counselling; benefits and housing assistance; acquired immunodeficiency syndrome (AIDS) videotapes and group sessions on AIDS education; HIV testing; condom use; use of bleach to sterilise injecting equipment; signposting to community resources; and tailored individual sessions.	1	Wright and Walker (2006) [66]

The included reviews varied in terms of their inclusion of populations of interest, with only a few focusing specifically on people who use drugs who reported being homeless [31, 61, 64, 70]. Others focused on people who were homeless and had co-occurring serious mental health problems and alcohol/drug use (COSMHAD) [62, 69] people who were homeless [59], or people who were homeless with mental health problems [56] as the primary population of interest, where substance use was secondary. Full details of the studies are presented in S3 Table.

There were notable differences in the proportion of participants who were homeless between the primary studies in the included reviews. For this reason some adopted minimum percentages for inclusion, for example Barker and Maguire [46] only included reviews when a minimum of 30% of included studies had a focus on homelessness, and Ponka et al. [63] required more than 50% of any study participants to be identified as ‘homeless’. The definition of homelessness also varied between the reviews, and between the included primary studies, which made it difficult to make direct comparisions between reviews.

### Treatment outcomes

The included reviews discussed a wide range of outcomes, including: those relating to substance use (reduction in drug and alcohol use (or tobacco); relapse rates; fatal and non-fatal opioid overdose rates; mean injecting frequency; and increase in treatment entry); housing; and ‘other’ outcomes, for example: well-being/quality of life (QoL); mental health; criminal justice system involvement; and societal integration. Four reviews [31, 62, 69, 71] grouped into ‘components of good practice’ focused on the elements of successful treatment rather than, or in addition to, investigating types of specific treatments. These outcomes have been synthesised below.

#### Treatment outcomes: Substance use

A variety of intervention types are available for people experiencing homelessness with concurrent problem substance use. These outcomes were reported in all 25 reviews, with mixed results overall. Regarding harm reduction interventions, these can lead to decreases in drug-related risk behaviour (e.g. needle sharing) for people who are homeless and use drugs [65], and co-delivery of a number of such approaches together (‘full harm reduction’) can lead to better outcomes than single harm reduction interventions. For example, full harm reduction, defined as receiving both opioid substitution therapy (OST) and high needle and syringe programme (NSP) coverage (100% versus <100% needles per injection), was associated with a 48% reduction in self-reported needle sharing, and in mean injecting frequency by 20.8 injections per month [65]. Wright and Tompkins [68] suggested that there was emerging evidence for the effectiveness of supervised consumption facilities (SCFs), as well as for peer distribution of take-home naloxone (THN), in reducing drug-related deaths for people who are homeless who inject drugs. Similarly, a recent study by Magwood et al. [60] concluded that SCFs decreased fatal overdose rates and reduced other high risk behaviours; and pharmaceutical interventions (such as OST) also reduced mortality, morbidity, and substance use [60]. Bates et al. [52] also concluded that OST led to reductions in drug use but, in contrast to Turner et al. [65], they did not find evidence of harm reduction interventions leading to a reduction in needle sharing.

For people with COSMHAD, Minyard et al. [53] presented some evidence for the effectiveness of an integrated day programme in reducing substance use rates, and Wright and Tompkins [68] reported that residential interventions led to greater reductions in drug use than community interventions. When comparing housing and support services with less intensive types of interventions, substance use outcomes were not significantly different [59]. However, there was some support for psychosocial rehabilitation, and an abstinence-contingent multifactorial housing programme with behavioural and work therapy interventions, in reducing substance use [59]. Moreover, there was support for education programmes in reducing injection drug use, specifically among homeless women [59].

Regarding housing interventions, the reviews suggested neither a positive nor a negative impact of HF on substance use, but it was deemed potentially helpful for stabilisation. For example, Pleace and Quilgars [54] reported no significant difference between HF participants and a control group in terms of either alcohol or drug use at 24- or 48-months post intervention in one of their included studies, with small but statistically significant improvements in alcohol and drug use over 24- months in another. Both Baxter et al. [2] and Beaudoin [55] found that HF produced no clear differences in substance use when compared with treatment as usual (TAU) which consisted of diverse alternative homeless services and interventions. Beaudoin [55] found no differences between those involved in HF interventions and those accessing traditional psychosocial interventions. However, Baxter et al. [2] reported that, in one of their included studies, participants housed together in dedicated accommodation blocks (single-site/congregate HF model) experienced greater improvements in problem substance use than those in scattered-site housing.

The evidence concerning permanent supportive and recovery housing (supportive housing promoting abstinence, specifically for those with alcohol or other substance use problems) [56, 57] respectively, also yielded mixed findings regarding substance use. Chambers et al. [57] found some evidence of the effectiveness of recovery housing and, although all evidence in their review stemmed from the USA, the authors suggested that the model could be replicated elsewhere (specifically the UK where the authors were based) and offered as an alternative to HF, allowing people to live in an abstinent community. Chambers et al. [57] concluded that recovery houses can improve personal well-being for some clients through promoting abstinence from alcohol or drugs.

Regarding case management interventions, Torres Del Estal and Álvarez [64] concluded that this type of intervention can lead to a reduction in substance use, either as a single intervention or in combination with others. De Vet et al. [58] provided some evidence that standard case management (SCM) is effective for people who are homeless and use drugs in reducing problem substance use, more so than TAU. Similarly, Ponka et al. [63] reported that SCM had both limited and short term effects on problem substance use, such as decreased problem substance use. Regarding assertive community treatment (ACT), findings were largely non-significant or inconsistent [58, 67]. Critical time intervention (CTI) was found to be significantly better than TAU in reducing substance use among people who were homeless with mental health problems, and intensive case management (ICM) led to substantial reductions in both drug and alcohol use [63].

Peer support interventions found some positive effects of intentional peer support (IPS), which is the type of peer support that is fostered and developed by professional organisations, on substance use, with an overall reduction in harm related to substance use, relapse rates, amount of money spent on substances, and number of days using drugs or alcohol [46]. Miler et al. [61] also reported a number of positive substance use outcomes relating to peer support, from both qualitative and quantitative studies. These included, for example, a significant reduction in mean daily cigarette use combined with a considerable reduction in self-reported illicit drug use, in a peer support smoking cessation study for people who were homeless with poly-substance use [61].

Lastly, Wright and Walker [66] examined the effectiveness of sexual health promotion interventions for people experiencing homelessness and using drugs, concluding overall that such interventions resulted in increased knowledge of drug-related harms and initially led to a reduction in drug use. Results regarding longer term effects (e.g. over a 24-month period) were mixed.

Overall, the evidence suggests that the more integration there is between programmes and services (as opposed to parallel service provision) when supporting people who have multiple needs, the better the outcomes. There is some evidence to suggest that harm reduction approaches can lead to decreases in drug-related risk behaviour, and to decreased fatal overdoses, as well as to reductions in all-cause mortality, morbidity, and substance use. Case management interventions, especially CTI and ICM, have been found to be significantly better than TAU in reducing substance use among people who were homeless, including those with mental health problems. Peer support interventions can have a positive impact on substance use outcomes. Lastly, the evidence regarding substance use outcomes and HF seems to indicate that HF does not lead to significant changes in substance use.

#### Treatment outcomes: Housing

Housing outcomes were reported in 10 of the included reviews [2, 46, 50, 55–58, 61, 63, 70].

Regarding HF, large improvements in housing stability were reported in one review, with intervention participants spending more days housed and more likely to be housed at 18–24 months post-intervention [2]. Similarly, Beaudoin [55] reported that HF resulted in more time spent in housing and less time on the street when compared with case management and TF programmes. Similarly, Kertesz et al. [70] concluded that, despite limited data, HF appears to improve housing retention in people experiencing homelessness and problem substance use. Moreover, Chambers et al. [57] found moderate-strength evidence for a positive effect of supportive housing on housing stability, including strong evidence that HF could improve housing stability. A range of factors which influenced the effectiveness of HF were identified, including fidelity to core components, and whether the service delivered a congregate or a scattered model. Lastly, Benston [56] found that most participants placed in permanent supportive housing programmes with case management, offered specifically to people who were homeless with mental health problems, remained in housing for at least 12-months, or experienced more days housed than homeless, relative to a comparison group.

Relating to case management interventions, there was some evidence that SCM was effective for people who were homeless and using substances in improving housing stability [58], and for having both limited and short term effects on housing outcomes [63]. On the other hand, for the same subgroup, findings regarding the effectiveness of ICM were mixed or inconsistent [58], with some small positive effects on housing outcomes and reductions in the number of days spent homeless, but no significant effect on the number of days spent in stable housing [63]. For people experiencing homelessness and mental health problems there was some evidence of positive effects of ICM on housing outcomes, and of CTI on housing stability [58]. Regarding ACT, de Vet et al. [58] found consistent improvements in housing stability for people with mental health problems, as well as those with COSMHAD, to a greater degree than less proactive case management models. Furthermore, Ponka et al. [63] reported both CTI and ACT to have a promising effect on housing stability, including more days in community housing, and fewer days homeless, and, in a US context, families that received CTI transitioned from shelter to housing more rapidly than the TAU group.

Emerging evidence suggests that peer support interventions for people who are homeless and use substances can lead to improved housing outcomes, including positive effects of IPS on the number of homeless days and return to homelessness [46]. Similarly, other peer support interventions for people experiencing homelessness with problem substance use can lead to positive housing outcomes, even if unintended, including improved housing in a smoking cessation peer support programme for people who are homeless with poly-substance use, or being supported to obtain housing by peers volunteering at safe injection/needle distribution sites [61].

Collectively, these reviews all support the HF approach in terms of its effectiveness in improving housing stability and retention. There is some evidence that supportive housing can also have a positive effect on housing stability. Peer support interventions have been found to lead to a decrease in number of days spent homeless, a reduction in return to homelessness, and other positive housing outcomes. A range of models of case management can be effective in improving housing outcomes, particularly for people experiencing homelessness and mental health problems, for whom ACT and CTI may be effective.

#### Treatment outcomes: Other

Sixteen of the included reviews examined outcomes other than housing or substance use [2, 46, 53, 55–61, 63–68], with health and well-being outcomes such as QoL and frequency of use of health services (including emergency departments, ED), as well as outcomes relating to crime, incarceration, and participation in community life.

Permanent supportive housing programmes yielded mixed mental health outcomes for people experiencing homelessness with mental health problems [56]. Similarly, the effects of HF on health and well-being outcomes were unclear in the short term, with no clear differences in terms of mental health or QoL compared with TAU [2]. However, HF clients showed a marked reduction in non-routine use of healthcare services over TAU which could be an indicator of improvements in health [2]. Similarly, largely non-significant or mixed results relating to the effects of HF on QoL were found, as well as for crime, incarceration, participation in community life, and victimisation [55]. Overall, HF does not seem to result in more positive effects on mental and physical health, and does not increase social support more than access to TAU, but there appears to be strong evidence that HF can improve measures of physical health in the short term for adults who are homeless or at risk of being homeless [57].

A range of complex interventions termed “other interventions for people with mental/physical health problems” [57] illustrate that these interventions provide an opportunity for recovery, but not everyone benefits. It was noted that some clients do not benefit or experience harmful effects, including social isolation and loneliness, when placed in single tenancy accommodation without adequate support [57] Moreover, interventions for specific groups of housing-vulnerable people presented largely mixed results regarding reductions in offending [57].

Reviews of case management interventions showed a positive effect of CTI on hospitalisation rates for people with problem substance use [58, 63], and a similar effect of ACT on client rehospitalisations [63, 67]. However, de Vet et al. [58] found that, while ACT influenced how people used mental health services, it did not appear to affect mental health outcomes. Additionally, CTI was found to be better than TAU in reducing mental health symptoms among those who are homeless with mental health problems [58]. CTI was also associated with shorter length of stays in hospital, and other institutional stays, coupled with achieving better long-term results than TAU, with similar associated costs [58]. Little evidence was found that SCM could lead to an increased use of services for people experiencing homelessness and problem substance use, with some evidence that SCM is effective for this group in removing employment barriers, but limited evidence of this for people who were homeless with COSMHAD [58]. Furthermore Ponka et al. [63] suggested that SCM can lead to increases rather than decreases in clients’ hostility and depression. The evidence base for ICM was limited, with largely non-significant or mixed findings, potentially partially due to treatment non-adherence [58].

Concerning programmes for people with COSMHAD, Hwang et al. [59] found that coordinated programmes for adults who were homeless with mental health problems or problem substance use generally resulted in better health outcomes than TAU, including mental health outcomes, and time spent in hospital. This was a finding similar to that of Minyard et al. [53], who found some evidence for the effectiveness of an integrated COSMHAD day programme for adults experiencing homelessness in reducing hospitalisation rates.

Regarding harm reduction interventions, both Turner et al. [65] and Magwood et al. [60] found that OST (and OST combined with high NSP coverage) can reduce the risk of contracting Hepatitis C (HCV), with the combined approach in Turner et al., [65] reportedly reducing the odds of new HCV infections by nearly 80%, as well as the risk of HIV infection. Findings on impact of OST on access to care were mixed [60]. Buprenorphine treatment was found to be associated with better access to treatment for patients not on methadone prescriptions, and patients who had began to use opioids more recently were able to access treatment earlier [60]. There was some evidence that frequent SCF use can be positively associated with experiencing a non-fatal opioid overdose within the SCF premises, and with a significant decrease in opioid overdose ED presentations, and with improved access to care for vulnerable populations [60]. SCF advantages included competent, non-judgemental staff, education on safer injection, and transfer to other medical (including hospitals) and social structures [60]. Furthermore, SCFs mediated referrals to services providing food and shelter and to other broader health support, as well as being associated with an increase in referrals to a problem substance use treatment centre and initiation of OST (in this case methadone maintenance therapy most specifically) [60]. Advice to seek treatment for an ongoing health condition by SCF staff was also associated with a significantly increased likelihood of receiving treatment [60]. No systematic reviews reported on the effects of SCFs on mental health outcomes.

Regarding peer interventions, Barker and Maguire [46] found that all included studies reported some positive effects of IPS in terms of overall QoL, mental/physical health, and increased social support. They also suggested that IPS works through components of shared experience, role modelling, providing social support, and increasing attendance/interest [46]. Similarly, Miler et al. [61] reported a number of positive outcomes in their review, such as changes in QoL and use of primary care, between baseline and six months, in a HF peer support study, and a range of psycho-socioeconomic benefits, including improvements in physical health, being able to return to work, and greater community engagement, in a peer support smoking cessation study for people who are homeless with poly-substance use.

Immunisation and smoking cessation programmes specifically for people who were homeless who used drugs resulted in positive health outcomes, including: smoking abstinence [59]; primary care utilisation in homeless families and children via outreach services [59]; and reduced subsequent ED visits as a result of compassionate care being provided from volunteers at ED presentation [59]. Moreover, sexual health promotion interventions for people who are homeless have the potential to improve psychosocial functioning [66]; and assertive outreach programmes for those with mental health problems, as well as informal programmes to promote sexual health, can lead to lasting physical and/or mental health gains [68].

Overall, there is some evidence that permanent supportive housing for people experiencing homelessness with additional mental health problems can lead to a reduction in mental health symptoms, and strong evidence that HF can improve measures of physical health in the short term. There is also evidence that integration of services and holistic treatment for people with COSMHAD leads to better psychosocial outcomes. Regarding case management interventions, ACT and CTI may be most promising for people who are homeless with substance use problems, given the positive effects on rehospitalisations, as well as reductions in mental health symptoms among those who are homeless with mental health problems. Moreover, harm reduction interventions including SCFs can lead to fewer hospitalisations and ED visits, and peer interventions can lead to changes in QoL and primary care use. There is also evidence that sexual health promotion interventions for people who are homeless have the potential to improve psychosocial functioning; and informal programmes to promote sexual health and assertive outreach programmes for those with mental health problems, can lead to lasting physical and/or mental health gains.

### Components of good practice

Four of the included reviews discussed components of good practice. Carver et al. [31] explored the views of people who used services and found that both harm reduction and abstinence-based treatments were considered effective but, in several studies, harm reduction-oriented services were preferred. However, clients also reported that abstinence-based treatments should be made available for when people are ready, highlighting that people who are homeless and experience problem substance use often desire an integrated approach to treatment. The review suggested that five components were important for effective treatment: i) the provision of a facilitative service environment; ii) compassionate and non-judgemental support; iii) adequate time in treatment; iv) choices regarding treatment; and opportunities to (re)learn how to live; and v) with these being delivered within the context of good relationships, person-centred care, and an understanding of the complexity of people’s lives. Longer treatment duration and stability was also valued, particularly by women [31].

Sun [71] reported four components of successful strategies for helping people who are homeless with COSMHAD: i) ensuring an effective transition for individuals with COSMHAD from an institution (e.g. hospital, foster care, prison, or a residential programme) into the community; ii) increasing the resources of people who are homeless with COSMHAD (e.g. helping them apply for government entitlements or supported employment); iii) linking individuals to supportive housing, including HF options, and being flexible in meeting housing needs; and iv) engaging individuals in treatment for COSMHAD. This includes incorporating modified ACT, motivational interviewing (MI), cognitive behavioural therapy, contingency management, and COSMHAD-specialised self-help groups.

Motivation for, and maintenance of, behaviour change was reported as a central factor for success in community-based services for people experiencing homelessness and COSMHAD [62]. Called ‘client choice’ in some programmes [62], this concept facilitated respect for the client’s treatment preference, even if this was not in line with what was considered the optimum treatment approach. Clients having input into staffing and programme elements resulted in a programme that was maximally tailored to their own needs, with data suggesting that both sense of mastery and perceived level of choice were mediators in the causal pathway between housing and a person’s psychiatric symptoms.

Provision of a more supportive, less intensive approach in residential programmes for people with COSMHAD was found to be a key to success [69]. Programmes rated by participants as being high in ‘support’, ‘involvement’, and ‘task orientation’, were associated with better outcomes, although it is not clear how these characteristics translated into specific programme components. In addition, specific modifications over the different stages of recovery, with a focus on slower, more concrete substance use counselling, flexibility in treatment, and greater support and guidance from staff, were also highlighted.

Collectively, these reviews suggest that flexibility is needed in treatment approaches, and that support should be tailored to the person. If possible, a combination of approaches should be used to offer choices to people who may not be ready for/do not want complete abstinence. Service providers need to be supportive and the treatment needs to be integrated, comprehensive, holistic, and person-centred, in order to increase effectiveness. Optimal duration also needs to be considered, with evidence suggesting that longer treatment leads to better outcomes, as well as being preferred by clients.

### Treatment entry, engagement, retention and successful completion

Twelve of the included reviews mentioned treatment engagement and/or retention [31, 39, 52, 54, 57–60, 67, 69–71] and six mentioned completion rates [46, 58, 59, 68–70], however, only one presented data as completion percentages [70], and one only provided completion percentages from one of the included studies [58].

There was some evidence of HF participants having higher rates of retention in a methadone treatment programme, compared with TF clients, and of increased engagement with medical treatment and mental health services. However, this was not the case for all clients, with identified barriers including boredom and isolation [57]. HF programmes were criticised in another review for a lack of engagement with services among those with very high levels of problem substance use, suggesting that TF could achieve better substance use outcomes, since they actively pursue abstinence from drugs and alcohol [54]. However, TF models have been reported to achieve relatively low rates of success, often losing between 40% and 70% of participants due to strict regimes, participants becoming ‘stuck’, or participants being evicted from services due to not meeting the abstention criteria [54]. One TF approach, called the ‘Birmingham model’, was found to lead to higher than average completion rates, with reports of 65% of participants completing a programme lasting 24 weeks [70].

Regarding case management approaches, de Vet et al. [58] noted participants not adhering to treatment and a lack of service use between groups in their included ICM studies. For example, 71% of participants assigned to shelter-based ICM services for men experiencing both substance use and homelessness did not complete the programme. On the other hand, Penzenstadler et al. [67] highlighted higher rates of treatment engagement and retention for ACT, as well as evidence of greater medication compliance, with significantly higher contact with patients in the ACT and integrated assertive community treatment (IACT) groups compared with controls. Overall, the authors concluded that ACT could be a promising approach that may be useful for promoting treatment engagement for people experiencing problem substance use.

Regarding harm reduction, findings on OST retention in treatment were mixed [60]. There does not appear to be any effect on treatment retention rates whether buprenorphine was administered under supervised or unsupervised criteria. However, methadone maintenance therapy was found to be more effective than non-pharmacological approaches in retaining heroin dependent patients in treatment, with no statistically significant difference in dropout rate between participants in slow release morphine versus methadone [60]. This suggests that the relative superiority of one pharmacological agent over another on retention outcomes remains unclear. Naltrexone implants showed significantly better treatment retention than placebo implants or oral naltrexone, and extended-release naltrexone led to significantly greater retention in treatment compared to TAU. However, successful completion of treatment rates did not differ when comparing oral naltrexone versus placebo [60].

Two studies included in Hwang et al.’s review [59] focusing on the treatment of latent tuberculosis (TB) for people who are homeless reported that, compared with TAU, a cash incentive increased attendance at an appointment for initial assessment of a positive tuberculin skin test. For people experiencing homelessness with latent TB, receiving directly observed preventive therapy, cash incentives, and non-cash vouchers at each visit were equally effective in increasing completion rates [59]. In other studies, there was some evidence that MI and motivational enhancement therapy (MET) increased treatment engagement in the short term for those experiencing homelessness and COSMHAD, and some evidence of benefits from the MI group in terms of increased attendance with aftercare [71]. Regarding engagement in treatment for people with HIV, Bates et al. [52] reported that adherence to highly active antiretroviral therapy (HAART) among people who used drugs was comparable to that among people who did not use drugs. However, people who used drugs and engaged in OST had increased adherence to HAART and better treatment outcomes, compared with people who used drugs who engaged in HAART alone.

For people with HIV, there was also evidence in support of the use of directly administered antiretroviral therapy, both alone and integrated in medication-assisted therapy, to improve treatment and outcomes related to blood-borne virus (BBV) infections. In terms of people with chronic HCV, there were no significant differences in BBV treatment dropout between people who inject drugs and those who do not who received combination treatment for HCV (ribavirin plus recombinant, or pegylated interferon-α). Lastly, for people experiencing homelessness who also injected drugs, an accelerated Hepatitis B immunisation schedule (with doses administered at 0, 7, and 21 days, and a booster at 12 months) resulted in superior completion rates, compared with traditional schedules with similar seroconversion rates [68].

Regarding peer support interventions, Barker and Maguire’s [46] review reported that their included IPS studies showed baseline data for 1,829 participants and completed data for 1,341 participants, with a loss to follow-up of 488 or 27% of participants. The authors [46] reported that one of the included studies suffered such extreme attrition from its control group that they excluded those data from the analysis, although the percentage dropout was not reported. This highlights challenges in retention in research studies for this group.

Overall, the evidence suggests that engaging and retaining people who are homeless and have substance use problems in treatment can be difficult, regardless of intervention type. There is evidence that ACT can lead to increased engagement rates for people who are homeless and use drugs, and that integrated services for people with COSMHAD lead to better engagement and retention than segregated treatments. Results regarding HF suggest that engagement can be difficult and that social isolation may be a problem for those using the service. Completion rates for the various treatment interventions are rarely reported, but tend to be low for case management interventions, especially for ICM.

## Discussion

We reviewed evidence from 25 reviews, published between 2004 and 2020, which explored the effectiveness of treatments and interventions for people experiencing homelessness and problem drug use. We examined the effects of these approaches on substance use, housing, and ‘other’ outcomes, as well as treatment entry, engagement, retention and completion, and components of good practice. A wide range of interventions were included, with evidence from specialist housing interventions, residential and community based programmes for people with COSMHAD, case management, abstinence-based and harm reduction oriented substance use treatment, healthcare interventions, peer support programmes, ED interventions, and sexual health promotion. The evidence regarding the effectiveness of these interventions is mixed. Integrated care for those experiencing homelessness and problem substance use, or COSMHAD, appeared to be associated with better outcomes. Harm reduction approaches had positive effects on drug-related risks, overdose, and other substance use outcomes, as well as on hospital visits and admissions. Case management, particularly ACT, CTI, and ICM, had positive effects on problem drug use, housing, and mental health outcomes. Housing interventions like HF improved housing stability and retention, and were associated with improvements in physical health, but had little effect on problem drug use. Relatedly, permanent supportive housing was effective for people experiencing COSMHAD in reducing poor mental health symptoms. Peer support interventions had positive effects on housing status and QoL, and sexual health interventions had positive effects on psychosocial functioning. Moreover, assertive outreach was associated with positive outcomes for people with COSMHAD in terms of their physical and mental health. Additionally, treatment approaches require to be flexible, person-centred, supportive, and integrated. Longer treatment duration, which offers a range of choices, is optimal. Engagement and retention is challenging, and assertive outreach and integrated care have the potential to reduce barriers to treatment.

It is important to ensure that those experiencing homelessness and problem drug use are provided with suitable healthcare, housing, and treatment. They are more likely to experience physical and mental health problems [19], and are at increased risk of drug related harms and early death than the general population [73, 74]. Access to health and substance use services can be challenging, often due to negative past experiences, discriminatory services, healthcare costs, and other administrative barriers [21, 22]. It is therefore important to understand the most effective ways of engaging and retaining people in services to ensure their needs can be met appropriately. The evidence regarding engagement and retention highlights the potential of peers and use of incentives with particular groups of people who are homeless who use drugs.

Taken together, this review highlights a range of interventions for a heterogeneous group of people with multiple complex needs: a ‘one size fits all’ approach does not exist for people experiencing homelessness and problem drug use. A range of approaches exist and it is likely that the approaches that are most effective are those which suit the particular needs of individuals, providing a range of options and addressing health, housing, and drug use in a holistic manner. Given the complexity of people’s needs and their varied experiences, the included reviews were not specific to people experiencing homelessness and problem drug use but also included, amongst others, people who are homeless with COSMHAD. This variability creates challenges in drawing conclusions on effective interventions for those experiencing both homelessness and problem drug use. However, our review does shed light on the types of interventions that are likely to be effective, the needs of particular sub-populations, and more general components of effective treatment.

### Policy, practice, and research recommendations

Our findings point to the need for a range of harm reduction oriented services to be available to those experiencing homelessness and problem drug use, including OST, NSP, SCFs, and peer distribution of THN. ‘Full’ harm reduction should therefore be made available to ensure people can access support without the expectation of abstinence. Additional work is also required to support those with BBVs through increased public health surveillance and research [65].

It is clear that the housing situation of individuals has a notable effect on their lives and should not be dictated by their substance use. Flexible and choice-led approaches to housing like HF may be beneficial, with more research required to identify the key components of HF and other approaches [54, 70]. Setting clear and realistic goals, particularly within the context of HF, is important, and services should recognise that achievable goals will differ between individuals [54]. This review has highlighted the potential of ACT, SCM, and CTI, and more research is required to compare these and other case management models in order to identify which models or specific components are most effective. Current treatment duration is often relatively short and there is evidence that extended treatment is associated with improved outcomes and perceived as beneficial [31, 75]. Therefore, further research is also required to identify the optimal length of treatment duration. Additionally, treatment requires suitable funding to ensure that it can continue for as long as necessary, so secure funding sources are also recommended. This is particularly important, but increasingly challenging, in the context of the COVID-19 pandemic, with already vulnerable services closing or restricting access [76, 77]. More research is also required regarding optimal policies on discharge planning for statutory agencies, which impact on continuity of care [78].

It is apparent that integrated care and partnership working are important aspects of providing services to people who are homeless [25]. Integrated mental health and problem substance use services appear to be particularly important for those experiencing homelessness and COSMHAD, with secure funding also required for such services [53]. However, more research is needed regarding such services in order to establish effective components of integrated programmes of support.

The way in which services are delivered appears to be vitally important, with compassionate and non-judgemental staff. It is therefore essential that services prioritise staff training to support them to gain an understanding of people’s complex lives, and the need for person-centered approaches, empathy and compassion. The context in which services are delivered is also crucial. For example, Pleace [39] noted the need for existing networks and support for joint working, and also to recognise the potential impact of: the availability and extent of welfare systems; social care and healthcare systems; general economic conditions; housing and labour markets; and waiting lists for social rented housing, on the effectiveness of interventions. Relatedly, involving peers in the delivery of services can be beneficial and more research is required to fully understand the effect of such individuals at the intersection of homelessness and problem drug use, as well as the impact of such services on peer workers themselves.

More qualitative research is required to understand people’s experiences of the various approaches, particularly from the viewpoint of sub-groups of people who are homeless with more complex needs due to their age, gender, ethnicity or sexual orientation/identity [31]. The heterogeneity of the populations and interventions included in this review point to the need for more research at the intersection between homelessness and problem drug use specifically, to ensure that the interventions for this group of individuals does meet their specific needs. While we can make suggestions regarding effectiveness, it would be misleading or inaccurate to base policy and service recommendations on evidence that is not specific to those experiencing homelessness and problem drug use.

### Strengths and limitations

Steps were taken throughout this review to enhance methodological rigour, including involvement of at least two people in literature searching, screening, quality appraisal, data extraction, and analysis. Including quantitative and qualitative reviews provided a more detailed understanding regarding the effectiveness of interventions, with insight into clients’ perspectives. We also included a range of international reviews, including two non-English reviews, to provide a detailed investigation of the topic.

Several limitations should be noted. Firstly, some of the included reviews were not systematic and were limited in their reporting on included studies, thus their findings should be interpreted with caution. Secondly, some of the reviews are relatively old, so the included studies are even older. The findings of these studies may be limited in terms of their relevance today, especially if no newer reviews have been conducted (e.g. [66]). Thirdly, while most of the reviews were international in focus, most primary studies were conducted in the USA or Canada, which may limit the transferability of the findings to countries where there are clear differences in terms of homelessness, healthcare, substance use and other related systems [79].

## Conclusion

People who experience both homelessness and problem substance use are a diverse group of people with complex lives and needs. Alongside dealing with the challenges imposed by homelessness, they are also simultaneously facing issues relating to their substance use. Many other social and health challenges are also likely to co-occur, such as mental health problems. There is a large evidence base regarding interventions for people who are homeless, and for people with problem substance use, but there is a lack of research focusing on the needs of people who experience both. Moreover, the evidence suggests that engaging and retaining people who are homeless and have substance use problems in treatment can be difficult regardless of intervention type, and completion rates for the various treatment interventions are rarely reported. Taken together, the findings from this review highlight the importance of integrating services to ensure a holistic and truly person-centred approach, as well as underlining the importance of *how* these interventions are delivered. We also highlight the need for a long(er)-term focus, including how individuals are ‘moved on’ into aftercare and what happens after formal treatment ends.

Overall, housing interventions, especially HF, have been the focus of much research, showing consistently positive findings regarding housing outcomes, but mixed results regarding health and well-being outcomes, with a lack of high-quality evidence on substance use outcomes. There is some evidence suggesting that harm reduction approaches can lead to decreases in drug-related risk behaviour, and to decreased fatal overdoses, as well as to reductions in all-cause mortality, morbidity, and substance use. There is mixed evidence regarding case management approaches, however CTI and ICM have been found to be significantly better than TAU in reducing substance use among people who are homeless, including those with mental health problems. ACT has also consistently reported positive effects on housing stability, and been found to be cost-effective, particularly for people with COSMHAD. Moreover, peer support approaches can lead to positive outcomes in housing, substance use, and well-being outcomes, as well as having the potential to have a positive impact on the peers themselves. However, care needs to be taken when embedding peers in services in order to ensure that they are respected, valued, and offered meaningful support and training opportunities.

## Supporting information

S1 PRISMA checklist (DOCX)

S1 Table Table of organisational websites searched. (DOCX)

S2 Table Quality appraisal table. (DOCX)

S3 Table Data extraction table. (DOCX)

S1 Data Search strategy. (DOCX)

S2 Data JBI critical appraisal checklist for systematic reviews and research syntheses. (DOCX)

S3 Data SANRA critical appraisal tool. (DOCX)

S4 Data Abbreviations list. (DOCX)
