# Supplementary material for: What treatment and services are effective for people who are homeless and use drugs? A systematic ‘review of reviews’
Source: PLoS One. 2021 Jul 14;16(7):e0254729. doi: 10.1371/journal.pone.0254729 (PMC8279330; doi:10.1371/journal.pone.0254729)
Supplement: S1 Table — (DOCX) [file pone.0254729.s002.docx]

**S1 Table. Table of organisational websites searched.**

| **Region** | **Name of organisation searched** |
| --- | --- |
| Scotland | Healthcare Improvement Scotland   Institute for Research and Innovation in Social Services  NHS Scotland  Salvation Army Centre for Addiction Services and Research online library  Scottish Drugs Forum   Scottish Government   Scottish Health Action on Alcohol Problems |
| Wider U.K. | British Psychological Society   Centre for Homelessness Impact  Crisis  Groundswell  Homeless Link  National Drug Evidence Centre  Pathway: The Faculty of Homeless and Inclusion Health  Public Health England  Royal College of Physicians  Royal College of Psychiatrists  Shelter  St Mungo's  The Salvation Army  We Are With You (formerly Addaction) |
| Republic of Ireland | Ana Liffey Drug Project  Crosscare  Depaul  Focus Ireland  Health Research Board  Health Service Executive Addiction Services   Merchant's Quay Ireland  Partnership for Health Equity  Peter McVerry Trust  Safetynet  Simon Communities  Threshold  YMCA Ireland |
| Europe | European Monitoring Centre for Drugs and Drug Addiction   European Observatory on Homelessness |
| USA | National Institute on Drug Abuse |
| Canada | Canadian Institute for Substance Use Research   Homeless Hub/Canadian Observatory on Homelessness |
| Australia | Centre for Social Research in Health  National Drug and Alcohol Research Centre |
| Global | United Nations Office on Drugs and Crime   World Health Organisation |
