# Supplementary material for: What treatment and services are effective for people who are homeless and use drugs? A systematic ‘review of reviews’
Source: PLoS One. 2021 Jul 14;16(7):e0254729. doi: 10.1371/journal.pone.0254729 (PMC8279330; doi:10.1371/journal.pone.0254729)
Supplement: S2 Table — (DOCX) [file pone.0254729.s003.docx]

**S2 Table. Quality appraisal table.**

| **Source** | **Clear research question** | **Appropriate inclusion criteria** | **Appropriate search strategy** | **Appropriate sources and resources** | **Appropriate criteria for appraisal** | **Appraisal conducted by 2 or more reviewers** | **Methods to minimize data extraction errors** | **Appropriate methods to combine data** | **Publication biased assessed** | **Recommen-dations for policy supported** | **Appropriate directives for new research** | **Total** |
| --- | --- | --- | --- | --- | --- | --- | --- | --- | --- | --- | --- | --- |
| Barker & Maguire (2017) | Yes | Yes | Yes | Yes | Yes | Not clear | Yes | Yes | Yes | Yes | Yes | 10/11 |
| Baxter et al (2019) | Yes | Yes | Yes | Yes | Yes | Yes | Yes | Yes | Not clear | Yes | Yes | 10/11 |
| Beaudoin  (2016) | Yes | Yes | Yes | Yes | Yes | Yes | Yes | Not clear | Not clear | Yes | Yes | 9/11 |
| Benston  (2015) | Not clear | Yes | Not clear | Not clear | No | No | Not clear | Yes | Not clear | Yes | Yes | 4/11 |
| Carver et al. (2020) | Yes | Yes | Yes | Yes | Yes | No | Not clear | Yes | Yes | Yes | Yes | 9/11 |
| Chambers et al. (2018) | Yes | Yes | Yes | Yes | Yes | Yes | Yes | Yes | Yes | Yes | Yes | 11/11 |
| De Vet et al (2013) | Not clear | Yes | Yes | Yes | Yes | Yes | Yes | Yes | Yes | Yes | Yes | 10/11 |
| Formosa et al  (2019) | No | Yes | Yes | Yes | Not clear | Not clear | Not clear | Not clear | Not clear | Not clear | Yes | 4/11 |
| Hwang et al (2005) | Not clear | Yes | Yes | Yes | Yes | Yes | Yes | Not clear | Yes | Yes | Yes | 9/11 |
| Magwood et al. (2020) | Yes | Yes | Yes | Yes | Yes | Yes | Yes | Yes | Yes | Yes | Yes | 11/11 |
| Miler et al. (2020) | Yes | Yes | Yes | Yes | Yes | Not clear | Yes | Yes | Yes | Yes | Yes | 10/11 |
| O'Campo et al (2009) | Yes | Yes | Yes | Yes | Yes | Not clear | Yes | Yes | Yes | Yes | Yes | 10/11 |
| Penzenstadler et al (2019) | Not clear | Yes | Yes | Yes | Yes | Not clear | Not clear | Not clear | Not clear | Yes | Yes | 6/11 |
| Ponka et al. (2020) | Yes | Yes | Yes | Yes | Yes | Yes | Yes | Yes | Yes | Yes | Yes | 11/11 |
| Torres Del Estal & Alvarez (2018) | No | Yes | Yes | Yes | Yes | Not clear | Not clear | Not clear | Not clear | Yes | Yes | 6/11 |
| Turner et al (2011) | No | Yes | Yes | Not clear | No | Not clear | Not clear | Yes | Not clear | Yes | Yes | 5/11 |
| Wright and Tompkins (2006) | No | Not clear | Not clear | Not clear | No | Not clear | Not clear | Not clear | Not clear | Yes | Yes | 2/11 |
| Wright and Walker (2006) | No | Yes | Yes | Yes | Yes | Not clear | Not clear | Yes | Not clear | Yes | Yes | 7/11 |

| **Source** | **Justification of the article’s importance for the readership** | **Statement of concrete aims or formulation of questions** | **Description of the literature search** | **Referencing** | **Scientific reasoning** | **Appropriate presentation of data** | **Total** |
| --- | --- | --- | --- | --- | --- | --- | --- |
| Brunette *et al.* (2004) | 2 | 1 | 1 | 1 | 1 | 1 | 7/12 |
| Kertesz *et al*. (2009) | 2 | 1 | 2 | 1 | 1 | 1 | 8/12 |
| Sun (2012) | 1 | 1 | 2 | 2 | 1 | 2 | 9/12 |
