# Supplementary material for: What treatment and services are effective for people who are homeless and use drugs? A systematic ‘review of reviews’
Source: PLoS One. 2021 Jul 14;16(7):e0254729. doi: 10.1371/journal.pone.0254729 (PMC8279330; doi:10.1371/journal.pone.0254729)
Supplement: S3 Table — (DOCX) [file pone.0254729.s004.docx]

**S3 Table. Data extraction table.**

| **Author (and organisation)** | **Title** | **Date** | **Location (country)** | **Summary of topic** | **Search strategy** | **No. of total included studies** | **Type of included studies (e.g. RCT)** | **Context/setting of interventions** | **Substance(s)** | **Population details** | **Intervention/treatment overview** | **Outcome(s) summary** | **Recommendations for policy and practice development** |
| --- | --- | --- | --- | --- | --- | --- | --- | --- | --- | --- | --- | --- | --- |
| **GREY LITERATURE REPORTS** | | | | | | | | | | | | | |
| Pleace (Centre for Housing Policy – University of York) | Effective services for substance misuse and homelessness in Scotland: Evidence from an international review | 2008 | The review was focused on countries broadly comparable with Scotland, including the European Union, other European countries, Australasia, Japan, and North America, and on England, Wales, and Ireland (including Northern Ireland). | An REA of international literature on effective substance use services for people who are homeless, to review best practice in other countries and determine if there were any lessons for Scotland. | REA. Two broad searches were conducted: one using the definitions agreed with the research advisory group; the second was broad and included all references that included the terms ‘Scotland’ and ‘homelessness’ alongside associated phrases. | Not specified. 6,645 references were screened. | Any study relevant to research question, with a focus on homelessness and substance use (this could be alongside other subjects), if not about Scotland, then had to be broadly applicable to Scotland. | Various (ranging from abstinence-based treatment to harm reduction) but all for people who use or had used drugs and had experience of homelessness. | All drugs (including alcohol). | People who use or had used drugs and who were at risk of homelessness, or who were homeless. Services were excluded from the review if clients did not meet these criteria. | 1) Joint working and case management (based on interagency working and delivering floating support to people in general needs housing). Support includes financial, training, employment, physical health, mental health, substance use, social and emotional support, and advocacy among others); 2) Fixed-site detoxification; 3) Staircase, continuum of care (CoC), and other transitional housing models; 4) The Pathways to Housing model; 5) Permanent supportive housing (all schemes take the form of shared, supportive housing, in which residents either have self-contained studio flats, bedsits, or rooms); and 6) Preventive services (including rent deposit schemes, housing advice, family reconciliation services, and various forms of debt counselling and financial management). | The review describes the difficulty with measuring outcomes of services, as it is not clear in the included literature which specific outcomes have been measured and project/programme outcomes differ significantly. Given its extent, the evidence base on case management and joint working models specifically related to homelessness and people who use drugs is surprisingly thin. The available USA research evidence indicates that fixed-set detoxification services tend to have limited success. There is little evidence on the effectiveness of single-site transitional housing in Scotland or elsewhere in the UK. HF models are largely viewed as more effective than the CoC model in the USA, but also have several conditions that are necessary to ensure that services could deliver good performance. There is not a significant evidence base relating to permanent supportive housing for people who are homeless with a history of problem substance use in the UK. It is not clear how successful these services are at harm reduction or supporting homelessness. In this review, the evidence on homelessness prevention services within Scotland is mixed. Prevention services identified also tended not to be focused on problem substance use. | 1) Realistic service outcomes need to be set and these should be tailored to individual clients; 2) The reason for treatment failure is often due to placing too many demands on clients, not providing the right range of support, and, in particular, not recognising the breadth and complexity of needs. Harm reduction models appear to meet with more success, even if the goals do not meet ‘abstinence’ goals. Outcomes including harm reduction, sustainable housing, improved quality of life, and generally increased stability can be achieved, even for highly vulnerable individuals with challenging behaviour; 3) Abstinence-based services can also be effective, even if only for a minority of homeless people with a history of substance use; 4) The evidence base suggests a need for a mixture of services; 5) Context needs to be taken into account. Outcomes in the service-rich environments of cities may be different from what is practical and achievable in smaller cities, towns, and more rural areas; 6) Outcome monitoring is essential to good service design and management. Longitudinal monitoring of service outcomes should be undertaken where possible; 7) The evidence base suggests that service interventions may need to be prolonged, creating a need for a secure funding base; and 8) Modification of generic services may be the best option in areas where numbers of people who are homeless and with a history of substance use are low. |
| Pleace and Quilgars (Centre for Housing Policy – University of York) | Improving health and social integration through Housing First: A review | 2013 | The review was international in scope. It included papers published in English as well as articles in French. | What happens after people who are homeless have been successfully rehoused? REA of the international evidence on success of HF in promoting healthcare integration and social and economic integration for people who were formerly homeless and using HF services. The REA covered service evaluations and research on HF services for people experiencing homelessness or potential homelessness, who are characterised by high support needs and recurrent or sustained homelessness. | REA. The searches for this review were carried out by a trained information specialist in the UK Centre for Reviews and Dissemination (CRD). Searches were run to identify the literature on the impacts of social, health, and community support in HF models. Firstly, a search of all ‘systematic research reviews’ on homelessness was carried out. A second, more extensive set of searches aimed to identify research of all study designs on community integration for people who are homeless and in supportive housing. | Not specified. 1,258 reviews were screened after deduplication. | The background search was for systematic reviews.  The second targeted search included all study designs. | All HF services, all of which provide people who are homeless with ordinary housing in the community. Treatment for drug use uses a harm reduction approach with a recovery orientation. | All drugs (including alcohol). | People who were formerly homeless and using HF services. | HF services follow a harm reduction framework with a recovery orientation. It is acknowledged that drug use occurs, and open use of alcohol is permitted. This tolerance of drug and alcohol use is accompanied by the provision of a range of support, either direct or delivered jointly with other services, to enable those in HF services to reduce, or stop, their substance use. The end goal of harm reduction services with a recovery orientation is the same as that for abstinence-based services – but rather than instruction to immediately cease all substance use, it seeks to create an environment where an individual can stop or reduce using substances when they choose to do so. | The available evidence on the effectiveness of HF services, from the main studies on HF, suggests that overall service utilisation is associated with stabilisation of drug and alcohol issues, rather than significant reductions or increases in drug and alcohol use. There is no evidence that drug and alcohol use increase following rehousing. | 1) Take lessons from other services; and 2) Enhancing HF in respect of health and social integration may involve recognising that different ‘levels’ of success will be achieved when promoting and enabling better health and social integration. Clear recognition that achievable goals may vary between each person using a HF service is the first step to becoming more precise about what HF services can achieve. Then, goals in relation to health and social integration outcomes should be defined, set, monitored, and tested using validated measures. |
| Minyard, et al. (Georgia Health Policy Centre) | Treatment services for people with co-occurring substance use and mental health problems. A rapid realist synthesis | 2019 | Undertaken in Ireland but with international focus. | Recent systematic reviews of effective treatments for COSMHAD are limited by their focus on specific mental health conditions or substances. They do not identify the contexts and mechanisms that may serve as facilitators for, or barriers to, achieving positive outcomes in providing integrated care. | A realist approach. A two-round iterative search. The searches were limited to results published between 1998 and 2018 which were written in English. | 151 (10,971 unduplicated articles were screened). | Any study type, but the 151 articles selected included 118 empirical studies (22 RCTs, 48 programme evaluations, 15 longitudinal analyses, 39 qualitative studies, 14 other); 16 syntheses or reviews (11 systematic reviews, 4 literature syntheses, 1 other); 16 brief reports; and 1 commentary. | Various interventions.  Articles were chosen, first, for their relevance to the research question. Only two studies with a homelessness focus were included. | All drugs (including alcohol). | People who use or had used drugs, and who were at risk of homelessness or who were homeless. | Integrated COSMHAD day programme. | Two of the included studies found an integrated COSMHAD day programme to be effective for the adult homeless population, reducing hospitalisation rates and decreasing substance use. | Policy/system: 1) Create incentives in payment to providers for integrating care of individuals with COSMHAD; 2) When developing service payment agreements, include deliverables that recognise the long path to recovery; 3) Analyse the system as it relates to access to psychological services and align providers with service needs; 4) Examine payment structure for peer mentors, coaches, and instructors; and 5) Explore how resources can be allocated to support a holistic approach to care (e.g. housing, supportive employment). |
| Bates et al. (Public Health Institute at Liverpool John Moores University) | The effectiveness of interventions related to the use of illicit drugs: prevention, harm reduction, treatment and recovery. A ‘review of reviews’ | 2017 | Undertaken in the UK but with an international focus. | To provide a synthesis of the best international research on responses to problem drug use. | Review of reviews. High-quality systematic reviews published since 2010 were identified through a comprehensive search of relevant electronic databases and screened for relevance against predefined inclusion and exclusion criteria. Lower-quality reviews and reviews published prior to 2010 were included where evidence was missing on key interventions. | 99 review articles divided under the headings ‘prevention’ (n=13), ‘harm reduction’ (n=24), and ‘treatment and recovery’ (n=62). | Reviews only. | Various interventions or treatments, not all relevant for people who are homeless. The ones that were relevant were community interventions for: adolescents who are homeless; people living with HIV who inject drugs; and people living with HCV who inject drugs. | All drugs (including alcohol). | Not specific to people who are homeless but did seek to highlight evidence on interventions for ‘high-risk’ groups, including people who are homeless and use drugs. Populations included adolescents, people with HIV who inject drugs, and people with HCV who inject drugs. | 1) Mentoring interventions: The review highlights the importance of the following interventions/treatment for people who are homeless due to increased risk of BBV in vulnerable populations; 2) HIV treatment approaches: highly active antiretroviral therapy (HAART), directly administered antiretroviral therapy (DAART), contingency management, and nurse-led interventions; 3) Harm reduction approaches for drug use and for needle sharing; and 4) Combination treatment approaches for HCV. | There was no benefit from a mentoring intervention delivered in combination with drug use treatment to adolescents who are homeless. Adherence to HAART among people who use drugs was comparable to among people who do not use drugs. People who used drugs and engaged in OST had increased adherence to HAART and better treatment outcomes than those receiving HAART alone. Evidence supported DAART alone and integrated in medication-assisted therapy to improve treatment and virological outcomes. Findings for contingency management are inconsistent but promising in favour of it. Findings for nurse-delivered interventions are inconsistent but promising. Individuals who received harm reduction approaches were significantly more likely to reduce drug use, but no significant differences in needle sharing between individuals who received harm reduction and controls were seen. | Because this review was not specific to people who are homeless, there were no specific recommendations in this area. However, the results in the outcomes summary column (see left) show what could work for future treatment/services for this population. |
| **ACADEMIC LITERATURE** | | | | | | | | | | | | | |
| Barker and Maguire  (University of Southampton) | Experts by Experience: Peer Support and its Use with the Homeless | 2017 | Undertaken in England, but had an international focus: 9 USA, 1 Canada, 1 Netherlands. | Attempting to understand what the literature reveals about IPS and homelessness. How IPS is currently being used with the homeless, the landscape of practice, outcomes of practice, and if IPS is a viable option for work with heterogeneous populations. Assessment of the effectiveness of IPS with a homeless population – peers are the intervention, not only delivering it. | A systematic review. Studies that had a primary intervention of IPS and its effects on those experiencing homelessness, with a minimum of 30% of the participants identifying as homeless. | 11 articles reporting on 10 studies. 4,028 articles screened after deduplication. Two articles reporting on the same dataset were combined for the purposes of this review. | Any type of study could be included, and the 10 studies comprise longitudinal, quasi-experimental, and cross-sectional studies. | IPS, where the peers are the intervention. | All drugs (including alcohol). | People who are homeless (at least 30% of each study’s sample), but did not look specifically at people who use drugs. Adults and young adults, including rough sleepers and those engaged in services. | IPS is termed ‘intentional’ because it is fostered and developed by professional organisations. IPS can be either mentorship support or mutual support; thus, studies that are using IPS may be using peers as client mentors or adjunct to services provided, such as combining peers and professionals in the delivery of services. IPS models are quite diverse; organisations not only utilise peers in multiple ways, but peers may or may not be trained and/or paid for their work. | All studies report some positive effects of IPS. Overall reduction in harm related to addiction observed. One-half of the studies report reduction in drug and alcohol use, and reducing relapse rates. Three studies report improvements on homelessness: decreases in number of homeless days, reduced relapse to homelessness, and reports of an overall improvement in environment. Positive outcomes relate to the improvement of the participants’ overall QoL; specifically, the reduction of drug/alcohol use, improved mental/physical health, and increased social support. | IPS can have a positive impact on outcomes for homeless people. Based on the evidence in this review, homeless organisations utilising peers should focus their outcomes on the areas where peers are shown to have impact, such as reduction of drug/alcohol abuse/use, increasing mental and physical health, and increasing social support. Practical applications of these results pertain to the training of peers, whereby training sessions focus on the common elements and the identified outcomes. For example, peers could learn how to use their shared experiences in a manner that models recovery from homelessness. |
| Baxter et al.  (University of Glasgow) | Effects of Housing First approaches on health and well-being of adults who are homeless or at risk of homelessness: systematic review and meta-analysis of randomised controlled trials | 2019 | Undertaken in Scotland with international focus. The included studies were all from the USA and Canada. | A policy approach aiming to end homelessness across Europe and North America – the HF model – provides rapid housing that is not conditional on abstinence from substance use. Authors systematically review the evidence from RCTs for the effects of HF on health and well-being. | A systematic search. Searches were restricted to studies published from 1992 up to the date of the search (15 May 2017) in peer-reviewed journals. Reference lists of previous reviews were checked for additional studies. The search strategy was developed in collaboration with a University of Glasgow librarian. Electronic databases were searched. | 25 eligible papers were identified for inclusion, reporting results from four studies. The four studies all had a high risk of bias. | RCTs of interventions providing rapid access to non-abstinence-contingent, permanent housing. Only studies published in English in peer-reviewed journals which reported a primary health outcome were included. | The intervention was defined as rapid provision of permanent, non-abstinence-contingent housing. The inclusion of additional supports (such as ICM or ACT) was not used to define the intervention. Compared interventions adhering with the wider principles of HF with interventions providing only housing; however, all studies included some form of additional support, so this subgroup analysis was not possible. All interventions included both rapid provision of permanent, non-contingent housing and additional support, thus are labelled ‘HF’ regardless of whether they were identified as such in the literature. | All drugs (including alcohol). | Adults (16 years and older) who meet at least one of the European Typology of Homelessness and Housing Exclusion (ETHOS) criteria: roofless, houseless, living in insecure housing, living in inadequate housing. Did not specifically look at substance use but 2/4 studies reported on this. | Providing access to housing through assistance in locating and entering housing, and subsistence of rental costs to maintain permanent tenancy. The housing provided was defined as intended to be permanent, counting sustained tenancy as the intended outcome. Not contingent on adherence to treatment or substance abstinence. Rapid, with the process of securing and entering housing initiated at first contact with the homeless person and with the aim of beginning tenancy promptly. | HF resulted in large improvements in housing stability, with unclear short term impact on health and well-being outcomes. For mental health, QoL, and substance use, no clear differences were seen when compared with treatment as usual (TAU). HF participants showed a clear reduction in non-routine use of healthcare services over TAU. This may be an indicator of improvements in health. Two studies reported substance use outcomes with mixed results. | HF approaches appear to be highly effective in reducing homelessness among vulnerable participants. However, in several direct measurements of short term health outcomes, the impact of HF is not clear. Further evidence could be valuable in assessing the long-term effects of improved housing stability on health. HF could be implemented with strong confidence in its success as a housing intervention, alongside some confidence in a lack of immediate adverse effects on health, but with caution in relying on this model for certainty in improved health outcomes. |
| Beaudoin (National Institute of Excellence in Health and Social Services (*Institut national d’excellence en santé et en services sociaux*; INESSS)) | Effectiveness of the “housing first” approach: A systematic review | 2016 | Undertaken in Canada with international focus; but majority of the included studies are from the USA. | The Pathways to Housing model has influenced the creation of several hybrid programmes, based on the HF approach combined with a range of services and diversified housing. This approach is based on the same philosophy as the original model: that people do not have to be abstinent or undergo psychiatric treatment to access accommodation. In Canada, the Government plans to primarily subsidise programmes based on this approach. | The search was carried out in several databases and in search engines on the Internet. January 2002 to January 2012 was the time frame within which results needed to have been published in order to be included, and an update was subsequently made, until June 2013. | 25 high-quality papers included. Quality of 39 assessed, and 14 of them were excluded due to their low quality. | Selection based on pre-established inclusion criteria for population, intervention, comparator, and results. Experimental studies, and quasi-experimental (with control group) and systematic reviews, were selected. 3 systematic reviews, 11 papers from 5 experimental studies, and 11 papers from 11 quasi-experimental studies were included. | Psychosocial interventions included traditional interventions, such as case management, and programmes based on the ‘treatment first’ approach. Housing voucher programmes were generally reserved for people who have first received treatment, who are abstinent, and whose mental health is stable, thus classified here as traditional psychosocial interventions. The HF approach refers to all the programmes and models in which housing support is offered without obligation of abstinence or compliance with prescriptions without treatment prior to access to housing. | All drugs (including alcohol). | Homeless people living with mental and/or substance-related disorders. | Programmes based on the HF approach and any type of HF-based programme with add-ons. | All the studies that have compared the HF approach with access to usual services show that participants in programmes based on the HF approach spend more time in housing and less time on the street. The results of the effects on QoL and satisfaction, crime, incarceration, participation in community life, and victimisation are mixed. Other results show that the HF approach does not have a more positive effect on mental health, physical health, substance use, or social support than access to usual services. | In the province of Quebec, HF types of programmes, typified by varying levels of housing and intervention intensity, should be promoted. However, they must take into account the ever-present dichotomy between the rural and urban realities of Quebec. |
| Benston. (State of Nevada Department of Health and Human Services) | Housing programs for homeless individuals with mental illness: Effects on housing and mental health outcomes. | 2015 | Undertaken in the USA with focus on USA. | This analysis reviewed results of the best available research in the USA on permanent housing programmes for homeless individuals with mental illness and the effect of these programmes on treatment outcomes, including housing status and mental health. | Limited to studies in which permanent housing was offered as an intervention and research input. A search of indexed literature from January 1980 through December 2013 was conducted, limiting ﬁndings to RCTs and quasi-experimental studies. | 12 primary studies and 2 secondary analyses, involving more than 7,400 homeless participants with mental illness. | Excluded research that did not include permanent housing (e.g. transitional housing, case management, outreach programmes). Studies that examined housing as an output, and studies involving homeless individuals who were not mentally ill or individuals with mental illness who were not homeless, were excluded. | Permanent supportive housing is loosely deﬁned as subsidised housing coupled with supportive services. No standardised model for housing homeless individuals with mental illness exists to guide research or policy. Housing studies involving this population are plagued by vague or conﬂicting deﬁnitions and conceptualisations of supportive housing, hampering comparative analysis. | All drugs (including alcohol). | Homeless individuals with mental illness. Did not look at substance use specifically but did include samples with substance use problems. | The reviewed studies deﬁned, designed, and implemented supportive housing in a variety of ways. Most of the studies did not speciﬁcally refer to their experimental housing conditions as ‘supportive housing’. The studies deﬁned and implemented case management in a variety of ways. Seven studies used ACT teams and others used services that included ICM, intensive clinical case management, and comprehensive case management, alongside traditional case management. | Majority of participants placed in experimental housing programmes with case management support remained in housing for at least one year or experienced more days housed than homeless relative to a comparison group. 7 of the 14 studies reported mixed clinical and substance use outcomes. One study reported that the experimental housing condition was associated with a reduction in substance use. Another study saw substance use decline in both conditions. Four studies found that the preferred housing condition did not yield any advantage in clinical outcomes over the comparison or control condition, either because no improvements were found or because both experimental and comparison groups showed similar gains. | There is a need for further experimental research to inform funding and policy decisions. The body of research is unable to answer fundamental questions about what type of housing programme works best for homeless individuals with mental illness. This review of the best studies on permanent supportive housing identiﬁed a small base of research with limited usefulness for decision-makers seeking empirical evidence to justify policy choices. The research cannot yet pinpoint which factors drive positive housing and clinical outcomes. |
| Brunette et al. (New Hampshire–Dartmouth Psychiatric Research Center and Dartmouth Medical School) | A review of research on residential programs for people with severe mental illness and co-occurring substance use disorders | 2004 | Undertaken in USA, with unclear focus. Studies from the USA and one from Honduras included. | Authors reviewed controlled studies of residential programmes for people with dual disorders (mental illness and substance use). | Searched electronic databases electronic databases and a variety of websites to also identify unpublished reports | 10 controlled studies of residential interventions. | Limited to controlled studies. | Residential programmes were recommended frequently for people with dual disorders, but their effectiveness was uncertain at the time. Authors categorised the interventions as short term (average stay <6 months) or long-term (>6 months), as longer length of participation may be associated with better outcomes. | All drugs (including alcohol). | People with COSMHAD. 5/10 studies focus specifically on people who are homeless. | Various long- and short term residential programmes. | 9 of 10 studies suggest advantages for integrated residential programmes that were modiﬁed to meet the needs of clients with severe mental illness (SMI), although each of these studies has major methodological issues. | Ultimately, a continuum of housing supports is necessary to serve people with dual disorders at different stages of treatment. |
| Carver et al. (Salvation Army Centre for Addiction Services and Research, University of Stirling, UK; and School of Health and Social Care, Sighthill Campus, Edinburgh Napier University, UK) | What constitutes effective problematic substance use treatment from the perspective of people who are homeless? A systematic review and meta-ethnography | 2020 | Undertaken in Scotland, focus is international. | People experiencing homelessness have higher rates of problem substance use but difficulty engaging with treatment services. There is limited evidence regarding how problem substance use treatment should be delivered for these individuals. Previous qualitative research has explored perceptions of effective treatment by people who are homeless, but these individual studies need to be synthesised to generate further practice-relevant insights from the perspective of this group. | Meta-ethnography.  Search in electronic databases for literature published between 2000 and 2019. Grey literature was identified by searching the websites of various relevant organisations for reports published since 2007. Reference lists of all included studies were reviewed. | 23 papers, involving 462 participants, were synthesised. 22 published papers and 1 grey literature study. Four papers were from two studies; thus, the findings from 21 studies were synthesised. | Qualitative, published between 2000 and 2019, plus grey literature. Eligible if: (a) reported primary qualitative research of perspectives of treatment for problem substance use; (b) was published in English; and (c) included adults aged 18 or over who were homeless/at risk of homelessness and had accessed treatment for problematic drug and/or alcohol use. | Any treatment for problematic drug and/or alcohol use. | All drugs (including alcohol). | Adults aged 18 or over who were homeless/at risk of homelessness and had accessed treatment for problematic drug and/or alcohol use (currently or in the 10 years prior to the study being conducted). Studies focusing on youth were excluded. | The 21 studies were conducted between 2002 and 2018 in the USA (n=11), Canada (n=7), and the UK (n=3), involving 462 participants. Participants were recruited from a range of services rather than directly from the streets. 10 studies provided insight into participant views of services generally, 1 study explored a hypothetical intervention, and 10 studies examined specific substance use interventions. Harm reduction and abstinence-based treatments featured in the reviewed studies. Interventions included Managed Alcohol Programmes and HF. | Participants in all types of interventions preferred harm reduction services and considered treatment effective when it provided a facilitative service environment; compassionate and non-judgemental support; time; choices; and opportunities to (re)learn how to live. Longer-duration n interventions that offered stability to clients were valued, especially by women. Critical components of effective substance use treatment include a service context of good relationships, with person-centred care and an understanding of the complexity of people’s lives. | Participants considered an effective intervention to be one which provided long-term treatment and ongoing support to help them to achieve stability. There is a need for development and evaluation of longer-term treatment and aftercare models, to avoid relapse, enhance stability, and enhance the likelihood of a range of positive outcomes, within both harm reduction and abstinence-based interventions. This desire from participants is in conflict with the reality of services globally, where austerity and systematic underfunding and cuts to services put pressure on services to discharge people as quickly as possible. |
| Chambers et al. (School for Health and Related Research (ScHARR), University of Sheffield, UK) | Systematic review of the evidence on housing interventions for ‘housing-vulnerable’ adults and its relationship to well-being | 2017 | Undertaken in the UK, focus is international. | Access to safe, good-quality, affordable housing is essential to well-being and housing-related factors can have an important influence on sense of community belonging. A recent scoping review on housing and well-being identified a lack of review-level evidence around the impact of housing interventions on well-being of people who are vulnerable to discrimination or exclusion in relation to housing. This systematic review was commissioned to address that gap. | Searched six bibliographic databases, performed reference and citation checking, and searched the websites of university departments and charities with expertise in housing.  Reviewed quantitative (experimental and observational) and qualitative research from the UK and other Organisation for Economic Co-operation and Development (OECD) countries published between 2005 and 2016. | 90 publications were included in the review. | Quantitative (experimental and observational) and qualitative research from the UK and other OECD countries published between 2005 and 2016. | HF (47)‚ other interventions for homeless people with mental health problems (11)‚ recovery housing (10)‚ supportive housing (12)‚ housing interventions for ex-prisoners (7)‚ housing interventions for vulnerable young people (3). | All drugs (including alcohol). | Inclusion criteria: Studies of housing-vulnerable adults, their families or carers, and providers of housing services. Had to include an intervention designed to avoid homelessness or unstable housing and report outcomes on well-being and/or housing stability. Housing-vulnerable people included those who were homeless or had a history of homelessness, people with a history of mental illness, people with a learning disability, refugees and recent immigrants, young people leaving care, and ex-prisoners. | HF (47)‚ other interventions for homeless people with mental health problems (11)‚ recovery housing (10)‚ supportive housing (12)‚ housing interventions for ex-prisoners (7)‚ housing interventions for vulnerable young people (3). | Strong evidence that HF can improve housing stability and measures of physical health in the short term. Evidence was classed as moderate for positive effects on personal well-being, mental health, and locality-related well-being (i.e. well-being related directly to one’s living situation and conditions) and for absence of effect on personal finance and community well-being. In other interventions for people with mental/physical health problems, a key finding was that they provide an opportunity for recovery but not everyone benefits. Recovery houses can improve personal well-being through promoting abstinence from alcohol or illegal drugs. Supportive housing found moderate strength of evidence for a positive effect on housing stability. However, strength of evidence for well-being outcomes was low or very low. | Findings may be difficult to translate into ‘actionable messages’ for policy and practice. Providing housing support for vulnerable people is clearly necessary but may not always be sufficient to improve their well-being and that of the community as a whole. In considering how to apply the evidence, decision-makers also need to take into account the wider context, including pressure on local authority budgets and changes in the political environment. |
| de Vet et al. (Department of Primary and Community Care, Radboud University Nijmegen Medical Centre, Netherlands; the Child Abuse and Neglect Team, Academic Medical Center, Amsterdam, Netherlands; and the Department of Orthopedagogics, Ghent University, Belgium) | Effectiveness of Case Management for Homeless Persons: A Systematic Review | 2013 | Undertaken by Dutch/Belgian team, focus international: 20 studies from USA, 1 from UK. | Reviewed the literature on standard case management (SCM), ICM, ACT, and critical time intervention (CTI) for homeless adults. | Electronic systematic literature search for peer-reviewed articles published in English between January 1985 and June 2011 in electronic databases. The title or abstract had to indicate that the study included an intervention. In the full-text article, at least one of the included interventions had to be identiﬁed as adhering to, or being based on, one of the four models of case management selected. | 33 publications pertained to 21 unique study samples. | RCTs or a before-and-after studies only, incorporating a baseline and at least one follow-up assessment of outcome variables, comparing two or more groups that received different interventions. The article had to include participant-level outcomes. | SCM, ICM, ACT, and CTI for homeless adults. | All drugs (including alcohol). | Participants in eligible study samples were aged 18 years or over. The recruitment strategy of the study had to target a predominantly homeless population, as evidenced by the description of the target population, recruitment setting, or selection criteria. Definition of homelessness provided. | SCM, ICM, ACT, and CTI for homeless adults. | Across the four different models, case management generally seemed to have a positive impact on housing stability and patterns of service use. Findings about substance use outcomes were mixed, and effects on variables measuring health, societal participation, and QoL were largely non-signiﬁcant. | To properly inform policy-makers in the European Union, experimental trials should be conducted among different homeless groups in a variety of service settings and countries. These studies should be carefully designed. They should aim for more uniformity in outcomes examined and for more standardisation of measurement instruments. Practitioners could employ case management to assist homeless persons with improving their housing stability and changing their service use patterns. |
| Form Farmosa et al. (NOT a study – conference abstract only; University Health Network, Toronto, Canada). | Emergency Department Interventions for Homelessness: A Systematic Review | 2019 | Undertaken in Canada; unsure about focus. | Study aims to aggregate and review the literature on emergency department (ED) interventions that improve health and/or access to the SDOH for homeless patients. | Developed search strategy with a library scientist, searching eight databases for peer-reviewed published studies as well as grey literature. Included studies of homeless patients (or majority homeless patients) that recruited patients at the level of the ED. | 13 studies. | Included studies had a control group or were a pre-/post-intervention trial, and measured outcomes that reflected the health (including access to the SDOH) of the studied subjects. | Various interventions included. | Drugs, but unclear if alcohol. | Included studies of homeless patients (or majority homeless patients) that recruited patients at the level of the ED. | Studied interventions included case management, HF, substance use interventions, ED-based resource desks, and ED compassionate care. | 8 studies (2 HF) sought to improve access to housing and 6 demonstrated significant reductions in homelessness and increased access to stable housing. 6 studies focused on homeless people using substances. 1 found extended release naltrexone feasible in a homeless demographic. 3 case management interventions reported reductions in alcohol and substance use and enrolment in substance use treatment. 1 study found no change in opioid overdose rates after opioid overdose education harm reduction initiative. | ED is in a unique position to try intervening in breaking the cycle of homelessness. While ED programmes that directly connect people with housing are beneficial, studies of ED-initiated case management can have similar results. |
| Hwang et al. (Centre for Research on Inner City Health, St. Michael’s Hospital, St. Joseph’s Health Centre, Toronto, Ontario; Departments of Medicine and Psychiatry, University of Toronto, Toronto, Ontario; and Dalhousie Medical School, Halifax, Nova Scotia, Canada) | Interventions to improve the health of the homeless: a systematic review | 2005 | Undertaken in Canada, focus is international. | This review evaluated interventions to improve health-related outcomes for homeless people. | Electronic databases were searched from inception to July 2004 for articles published in English. The bibliographies of relevant reviews and included articles were screened for additional studies. | 73: 37 RCTs, 19 prospective longitudinal studies, 10 retrospective studies, and 7 secondary analyses of RCT data. | RCTs, prospective longitudinal studies with non-randomised allocation to different treatment groups, retrospective studies with comparison of outcomes in different treatment groups, and secondary analyses of RCT data where the intervention was not the same as that allocated in the original trial. | Interventions for people with mental illness (15 studies). Interventions for people with substance use (13 studies). Interventions for people with concurrent mental illness and substance use (7 studies). | All drugs (including alcohol). | Studies of homeless people, including those with no fixed, regular, and adequate night-time residence, and those living in supervised shelters or locations not intended for human use. Studies that included non-homeless people as part of the population had to include at least half who were homeless and report the results separately. | Studies of any intervention provided by primary care, or to which homeless patients could be referred. The eligible comparators were another intervention or no intervention (usual care). Those included in the review were case management services and/or supportive housing; ACT; the Access to Community Care and Effective Services and Supports programme; post-detoxification stabilisation; abstinence-contingent work therapy; intensive residential treatment; other preventive health interventions for substance dependence; cash incentive schemes; educational programmes; and outreach initiatives. | Coordinated programmes for homeless adults with mental illness or substance use generally result in better health outcomes than usual care. Cash incentives were effective in increasing adherence to tuberculosis screening and improved completion rates; education initiatives improved HIV risk behaviours in runaway youth; outreach services reduced primary care utilisation in homeless families and children; education programmes reduced injection drug use among homeless women; and compassionate care reduced ED visits. | Practice: The authors stated that clinicians should focus on directing homeless people to tailored, coordinated treatment and support programmes.  Research: The authors stated that future studies should include usual care control groups and address the diversity of the homeless population, with focus on the needs of runaway youth and homeless families and children. |
| Kertesz et al. (Center for Surgical, Medical Acute Care Research and Transitions at the Birmingham Veterans Affairs Medical Center, University of Alabama at Birmingham, USA) | Housing First for homeless persons with active addiction: are we overreaching? | 2009 | Undertaken in the USA. Focus unclear but possibly USA. | The authors compare the effectiveness of HF for homeless people who have ‘addiction disorder’ with linear approaches on a number of outcomes. | Primarily comparative studies of HF and linear approaches to homeless persons with addiction and with or without concurrent non-addictive mental illness through electronic databases, Google Scholar, websites, and a review of health-oriented studies. Articles published in English in peer-reviewed journals. | Not reported. | (1) The target population (homeless, with addiction or mental illness); (2) Use of quantitative data; (3) A comparative study design with randomised or pseudorandomised design assessing a linear or HF approach; and (4) Inclusion of housing outcomes. Several non-comparative studies (e.g. case series) were included if they had illustrative value not available through comparative research. | More than 350 communities in the USA have committed to ending chronic homelessness. One nationally prominent approach, HF, offers early access to permanent housing without requiring completion of treatment or, for clients with addiction, proof of sobriety. | All drugs (including alcohol). | Homeless individuals with addictive disorders, with or without concurrent non-addictive mental illness. | Various HF interventions. The ‘linear’ approach anticipates that homeless persons with varying disabilities will enter rehabilitation-oriented programmes with the long-term goal of returning to housing. The linear approach generally makes rehabilitative treatment, typically residential, a prerequisite to permanent housing. Subsidised permanent housing depends on the client’s success in the programme, which typically requires abstinence from drugs and alcohol. | HF reports document excellent housing retention, despite the limited amount of data pertaining to homeless clients with active and severe addiction. Several linear programmes cite reductions in addiction severity but have shortcomings in long-term housing success and retention. | This article suggests that the current research data are not sufficient to identify an optimal housing and rehabilitation approach for people who are homeless and use substances. The research regarding HF and linear approaches can be strengthened in several ways, and policy-makers should be cautious about generalising the results of available HF studies to those with active substance use when they enter housing programmes. |
| Magwood, et al. (Bruyère Research Institute, Ottawa, Canada, Department of Family Medicine, University of Alberta, Edmonton, Canada, St. Michael’s Hospital, University of Toronto, Canada, McGill University, Montreal, Canada, Public Health and Preventative Medicine Residency Program, University of Ottawa, Canada, McMaster University, Canada | The effectiveness of substance use  interventions for homeless and vulnerably  housed persons: A systematic review of  systematic reviews on supervised  consumption facilities, managed alcohol  programs, and pharmacological agents for  opioid use disorder | 2020 | Undertaken in Canada; international focus | A systematic overview of reviews examining the effects of selected harm reduction and pharmacological interventions (supervised consumption facilities, managed alcohol programs, and pharmacological agents for opioid use disorder) on the health and social well-being of people who use substances, with a focus on homeless populations. | Systematic search using relevant keywords and MeSH terms for relevant published systematic reviews. Keywords included terms such as “opioid-related disorders”, “supervised consumption”, “supervised injection”, “managed alcohol”, “methadone” and “harm reduction”. Searched electronic databases from database inception to August 2019. There were no language restrictions. Grey literature searched. Included all outcomes of interest including: mortality, morbidity, substance use, mental health, access to care, and retention in treatment. | 30 systematic reviews met all inclusion criteria, capturing the results from 442 primary studies. | Included systematic reviews of quantitative or qualitative studies that focused on at least one of the interventions specified in protocol among homeless or general populations. Any type of comparator was considered eligible (such as usual services, alternative intervention or no intervention). Excluded overviews of reviews and narrative literature reviews | Harm reduction only. Selected reviews that synthesized evidence on supervised consumption facilities, managed alcohol programs and pharmacological interventions for opioid use disorders. Reviews on pharmacological interventions reported on the use of methadone, buprenorphine, diacetylmorphine (heroin), levo-α-acetylmethadol (LAAM), slow release oral morphine and hydromorphone for treatment of opioid use disorder. One review reporting the effectiveness of naloxone among populations experiencing opioid use disorder, which identified four trials that investigated naloxone in the treatment of suspected or established opioid overdose. Supervised consumption rooms and MAPs. | Drugs and alcohol | People who are homeless and use substances | Harm reduction – opioid substitution treatment; safe consumption facilities (SCFs); naloxone programmes for overdose prevention or reversal; and managed alcohol programmes (MAPs). | Review found that harm reduction interventions reduce harms associated with substance use and mitigate morbidity and mortality. Several studies on pharmacological interventions demonstrated improved outcomes for mortality, HCV and HIV acquisition, psychological morbidity and non-prescribed opioid use. Results suggest that buprenorphine and methadone are the most effective pharmaceutical agents to address mortality and morbidity among people who use substances. Safe consumption facilities had positive impacts on fatal overdose prevention, public injecting and other high-risk consumption, and can act as a bridge to other health and social services. | More high quality research is required on MAPs, the scaling up of SCFs in a variety of settings such as supportive housing environments or as part of healthcare centres, as well as the types and ideal prescribing strategies of pharmacological interventions. Longer follow-up periods are also required to determine which interventions maintain their improvements over time. In order to inform decisions on resource allocation by public health and governmental bodies, future studies should continue to assess the cost effectiveness of these interventions by evaluating and comparing the cost of their implementation against their benefits at the individual and societal levels. |
| Miler, et al (Salvation Army Centre for Addiction Services and Research, University of Stirling, Scotland, UK) | Provision of peer support at the intersection of homelessness and problem substance use services: a systematic ‘state of the art’ review | 2020 | Undertaken in Scotland, international in focus | Peer support refers to a process whereby individuals with lived experience of a particular phenomenon provide support to others by explicitly drawing on their personal experience. It has been adopted in a variety of service contexts including homelessness, substance use, mental and physical health. Those who experience homelessness have some of the most complex intersecting health and social challenges. This ‘state of the art’ review provides a systematic search and synthesis of literature examining use of peer support models within services for people impacted by homelessness and problem substance use. | A systematic search using six databases conducted in August 2019 identifying papers published in English after the year 2000. | 62 papers included studies conducted in five countries | methodologically diverse included a systematic reviews, qualitative, quantitative and mixed methods studies, and grey literature reports | Interventions ranged from interventions targeting specific populations, for example peer support with individuals with HIV or criminal justice involved/experienced individuals to focusing on specific harm reduction interventions or practices, for example needle exchange programmes or safe consumption sites. The largest number focused specifically on peer interventions with individuals experiencing homelessness. | Drugs, alcohol and tabaco | People who are homeless and use substances | Harm reduction (including needle/syringe exchange; safe/supervised injection consumption sites; naloxone training/distribution; Abstinence-based programmes including AA/12 Step; relapse; and recovery; smoking cessation interventions; Physical health including interventions for people with Tuberculosis, Hepatitis; and HIV | In recent years there has been a substantial increase in research examining the utility of peer support yet there is significant variation across this field. Alongside profiling the range of settings, aims, populations, and main outcomes of these studies, this paper also provides an overview of overarching themes: the overall effectiveness and impact of peer-staffed or peer-led interventions; and challenges commonly faced in these roles. Five themes relating to the challenges faced by peers were identified: vulnerability, authenticity, boundaries, stigma, and lack of recognition. | The findings provide support for current efforts to involve individuals with lived experience in providing peer support to those experiencing concurrent problem substance use and homelessness, but they also urge caution because of common pitfalls that can leave those providing the support vulnerable. Authors conclude that peers should be respected, valued, supported, and compensated for their work which is often profoundly challenging. Suggested guidelines for the implementation of peer involvement in research studies and service delivery are presented. |
| O’Campo, et al. (The Centre for Research on Inner City Health, The Keenan Research Centre, LiKaShing Knowledge Institute, St. Michael’s Hospital, Toronto, Canada; Dalla Lana School of Public Health, University of Toronto, Canada; Department of Psychology, Ryerson University, Toronto, Canada; Johns Hopkins Bloomberg School of Public Health, Baltimore, USA.) | Community-based services for homeless adults experiencing concurrent mental health and substance use disorders: a realist approach to synthesizing evidence | 2009 | Undertaken in Canada, focus is international. | Collaborative research effort between academic-based and community-based partners to conduct a realist review focusing on both whether programmes are successful and why and how they lead to improved outcomes. Addressing a gap in knowledge regarding effectiveness of programme components in community-based treatment approaches for homeless people with concurrent mental health and substance use disorders in urban community settings. | Authors present a list of all searched databases and supplement scholarly literature with grey literature. Authors also emailed corresponding authors of the included studies to learn more about how each programme operated. | 17 scholarly articles included (corresponding to 10 programmes) supplemented by other sources; overall, included 38 sources of evidence on the 10 programmes. | All English literature since 1980 with keywords related to interventions and mental health and substance use disorders was considered. | Treatment of concurrent mental health and substance use disorders has emerged only since the early 1990s with literature ospitalizati by poor evaluation designs, short term follow-up, and heterogeneous interventions and populations. Several types of interventions lack enough evidence to determine their effectiveness. Case management has been met with inconsistent findings. | All drugs (including alcohol). | Homeless individuals with concurrent mental health and substance use disorders. | Authors identified 10 distinct community-based or community-linked programmes serving homeless individuals experiencing concurrent mental health and substance use disorder that employed a variety of approaches including ACT, provision of housing, integrated mental health and substance use treatment, and a holistic approach through which many of the clients’ life needs were supported. Most programmes delivered a combination of programme strategies or took different approaches to the same strategies. | Authors identified six important and promising programme strategies that reduce mental health and, to a far lesser degree, substance use problems: client choice in treatment decision-making, positive interpersonal relationships between client and provider, ACT approaches, providing supportive housing, providing supports for instrumental needs, and non-restrictive programme approaches. | Continuous involvement of community-based agencies in various stages of the research process. While experience and expertise from the community partners was key in the integration of knowledge, the evidence gathering process, as well as the extraction and synthesis phases, the authors were particularly motivated to retain involvement of these key stakeholders to maximise the chances that the evidence will be used to change or inform current practice or policy. |
| Penzenstadler et al. (Geneva University Hospitals, Switzerland; Geneva University, Faculty of Medicine, Oxford Health National Health Service (NHS) Foundation Trust, UK; and Research Center, Montreal University Institute of Mental Health, Quebec, Canada) | Effect of Assertive Community Treatment for Patients with Substance Use Disorder: A Systematic Review | 2019 | Undertaken by a collaborative team from Switzerland, the UK, and Canada; focus is international. Most of the studies originate from the USA and one from the UK. | The ACT model was originally developed for patients with a severe mental illness but has been adapted for patients with substance use disorder (SUD) by integrating specific SUD treatments into the traditional ACT model. This paper aims to assess the effectiveness of ACT for patients with SUD on several measures. | Authors performed a systematic search.  No grey literature search. No mention of contact with experts or looking at references list. | 11 publications using 5 datasets were included in the analysis. | RCTs published before June 2017 found on the electronic databases. | The key elements of the ACT model are assertive engagement, delivery of services in the community, high intensity of services, holistic and integrated services by multidisciplinary teams, and continuity of care. In order to provide a high intensity of care, the caseloads are small and in the original model, a 24-hour service is provided. | All drugs (including alcohol). | Patients included in the studies had a diagnosis of SUD. Two datasets included homeless patients and two datasets included patients with high service use. | The services in the included studies all used the principles of ACT in their approach, with services provided in the community, assertive engagement, high intensity of services, small caseloads, 24-hour responsibility, a team approach, and multidisciplinary working. | The results of the very few existing RCTs are mixed. Treatment engagement was higher for ACT in four datasets. One dataset reported higher service contact rates for the ACT group than for controls. In two datasets a positive effect on ospitalization rates was found. Higher fidelity to the ACT model appears to improve outcomes. Substance use reduced only in half of the datasets, of which only one showed a significant reduction in the ACT group. | The research base is variable concerning the usefulness of ACT in the field of addiction. Higher fidelity to the ACT model appears to improve results and studies often found at least one outcome measure improved. Future research should investigate the effective ‘ingredients’ of ACT. This would help to conceptualise a specific ACT model that may be more effective. Further research is needed to examine which types of clinical interventions might help difficult-to-engage patients with addictions in order to innovate treatment approaches and reach out to patients. |
| Ponka, et al. (University of Ottawa, Canada; University of Roehampton, London, United Kingdom; Bruyère Research Institute, Ottawa, Canada; , Department of Psychiatry, University of Toronto, Canada; University of Calgary, Canada; , Wilfred Laurier University, Canada; , McGill University, Canada) | The effectiveness of case management interventions for the homeless, vulnerably housed and persons with lived experienc: A systematic review | 2020 | Undertaken in Canada with a UK collaborator. International focus. | Standard case management interventions as well as more intensive models with practitioner support, such as assertive community treatment, critical time interventions, and intensive case management, may improve healthcare navigation and outcomes. However, the definitions of these models as well as the fidelity and adaptations in real world interventions are highly variable. Authors conducted a systematic review to examine the effectiveness and cost-effectiveness of case management interventions on health and social outcomes for homeless populations. | Searched electronic databases from the inception of these databases to July 2019. Authors sought outcomes on housing stability, mental health, quality of life, substance use, hospitalization, income and employment, and cost-effectiveness. | 56 primary studies met full inclusion criteria. | Primary studies; Randomized controlled trials Non-randomized controlled trials Controlled before-after studies Interrupted time series and repeated measures studies Cost or cost-consequence studies Full economic evaluation studies: cost-minimization analysis, cost-benefit analysis, cost-effectiveness analysis, and cost-utility analysis. All study designs must include interventions with a comparison/ control group and have measured outcomes. | People who are homeless or vulnerably housed may benefit from tailored, patient-centered care with an integrated approach to community and social services. Case management (CM) is one such intervention where individual case managers respond to the complexity of navigating the healthcare system by assessing, planning and facilitating access to health and social services. While case management interventions are heterogeneous in definition, complexity, target populations served, and modes of delivery, among these, four predominant models have evolved in relation to health care: standard case management (SCM), intensive case management (ICM), assertive community treatment (ACT), and critical time intervention (CTI) | Drugs and alcohol | People experiencing homelessness and vulnerable housing. If study populations were heterogeneous, authors included the study if the population was comprised of >50% homeless or vulnerably housed individuals. | Standard Case Management Intensive Case Management Assertive Community Treatment Critical Time Intervention | Standard case management had both limited and short term effects on substance use and housing outcomes and showed potential to increase hostility and depression. Intensive case management substantially reduced the number of days spent homeless as well as substance and alcohol use. Critical time interventions and assertive community treatment were found to have a protective effect in terms of rehospitalisations and a promising effect on housing stability. Assertive community treatment was found to be cost-effective compared to standard case management. | Case management approaches were found to improve some if not all of the health and social outcomes that were examined in this study. The important factors were likely delivery intensity, the number and type of caseloads, hospital versus community programs and varying levels of participant needs. More research is needed to fully understand how to continue to obtain the increased benefits inherent in intensive case management, even in community settings where feasibility considerations lead to larger caseloads and less-intensive followup. |
| Sun (School of Social Work, University of Nevada, Las Vegas, Nevada, USA) | Helping Homeless Individuals with Co-occurring Disorders: The Four Components | 2012 | Undertaken in the USA, focus is on USA. | Homeless individuals with COSMHAD are one of the most vulnerable populations. This article provides practitioners with a framework and strategies for helping this client population. | Database searches plus the reference lists of located articles. | Not reported. | All study types. | Various interventions. | All drugs (including alcohol). | Homeless individuals with COSMHAD | Various interventions, e.g. CTI (an evidence-based treatment that goes one step beyond a discharge plan, where during the ﬁrst months after discharge, when a client’s relationship with people in the community may be fragile, CTI strengthens the client’s adjustment to the community by pairing the client with a social worker who visits the client’s community residence, accompanies the client to appointments, and helps the client develop relationships with people at the appointments and provides advice in periods of crisis); Motivational Interviewing (MI) before client discharge (the MI session addresses the differences between hospital and outpatient treatment regarding the treatment goals and methods and engages the client to explore his or her own understanding of his or her clinical condition and commitment to treatment). | Four components emerged from a literature review: (1) ensuring an effective transition for individuals with COSMHAD from an institution; (2) increasing the resources of homeless individuals with COSMHAD by helping them apply for government entitlements or supported employment; (3) linking homeless individuals to supportive housing, including HF options as opposed to only treatment ﬁrst options, and being ﬂexible in meeting their housing needs; and (4) engaging homeless individuals in COSMHAD treatment, incorporating modiﬁed ACT, MI, cognitive behavioural therapy (CBT), contingency management, and COSMHAD specialised self-help groups. | This article suggests four components: (1) ensuring effective transition of homeless individuals from institutions into community living; (2) helping them apply for government entitlements and obtain supported employment; (3) linking them with supported and supportive housing; and (4) applying and combining modiﬁed ACT, clinical case management, MI/Motivational Enhancement Therapy, CBT, contingency management, and specialised 12-step groups to maximise treatment effects. |
| Torres Del Estal, Álvarez (Hospital Universitario La Paz, Madrid, Spain) | *Intervenciones enfermeras para el manejo de adicciones a sustancias químicas de personas sin hogar* (Nursing interventions for the management of substance use of homeless persons) | 2018 | Undertaken in Spain, focus is international. Studies in English, Spanish, and French searched. | Substance use is one of the most prevalent health problems in people who are homeless. The aim is to review the literature about the effective nurse-led interventions for the management of substance use in people who are homeless. | Electronic databases were searched. Quantitative design studies (primary and secondary) and mixed methods studies, published in English, Spanish, and French in the last 10 years, were included. | 15 studies included. | Quantitative design studies (primary and secondary) and mixed, methods studies,published in English, Spanish, and French in the last 10 years. | Nurse-led interventions were classified according to the intervention model: case management or other nurse-led intervention models. | Drugs (not alcohol). | Homeless people who use drugs. | Case management versus other nurse-led intervention models. | Case management is an option with effective results either as a single intervention or in combination with others. Programmes that combine case management and interventions carried out in the context of interdisciplinary teams (nursing, medicine, psychology, and public health) have better results than standard interventions. | Nursing must be involved in programmes that develop feasible and effective interventions that reduce the health problems in people who are homeless. Apart from being effective alone, it could be accompanied with good results by other interventions such as contingency management with positive reinforcement or incentives, artistic therapy, health prevention and promotion programmes, and multidisciplinary teams. |
| Turner et al*.* (University of Bristol, UK; Health Protection Scotland, UK; University of Strathclyde, UK; London School of Hygiene and Tropical Medicine, UK; Health Protection Agency, Centre for Infection, London, UK; Health Protection, Public Health Wales, UK; University of the West of Scotland, UK; Institute of Public Health, Cambridge, UK; and West of Scotland Specialist Virology Centre, UK) | The impact of needle and syringe provision and opiate substitution therapy on the incidence of hepatitis C virus in injecting drug users: pooling of UK evidence | 2011 | Collaborative study by various researchers across the UK, focus is UK. | To investigate whether opiate substitution therapy and needle and syringe programmes (NSPs) can reduce hepatitis C virus (HCV) transmission among PWID. | Studies were included if they contained individual-level data on both intervention coverage (NSP and/or opiate substitution therapy) and a measure of newly acquired HCV infection among PWID surveyed in the community. Consulted UK experts and reviewed electronic databases. Studies published prior to 2000 or conducted in prisons were excluded. | Six UK studies with participants numbers as follows: Birmingham (n=310), Bristol (n=299), Glasgow (n=947), Leeds (n=302), London (n=428), and Wales (n=700). | Included if contained individual-level data on both intervention coverage (NSP and/or opiate substitution therapy) and a measure of newly acquired HCV infection among PWID surveyed in the community. Studies published prior to 2000 or conducted in prisons were excluded. Four cross-sectional studies (Birmingham, Bristol, Glasgow, and Leeds) and two cohort studies (London and Wales). | Opiate substitution therapy and NSPs. | Injection drugs. | People who inject drugs. Did not look at homeless people specifically, but each study sample contained between 32% and 62% of people who were homeless in the previous year. | Two important interventions for PWID are opiate substitution therapy to reduce drug dependence and injecting frequency, and the provision of clean injecting equipment through NSPs to reduce unsafe injecting (i.e. sharing used syringes). | Both receiving opiate substitution therapy and high NSP coverage were associated with a reduction in new HCV infections. Full harm reduction (on opiate substitution therapy plus high NSP coverage) reduced the odds of new HCV infection by nearly 80% (adjusted odds ratio (AOR)=0.21, 95% confidence interval (CI): 0.08–0.52). Full harm reduction was associated with a reduction in self-reported needle sharing by 48% (AOR=0.52, 95% CI: 0.32–0.83) and mean injecting frequency by 20.8 injections per month. | NSP, especially if combined with opiate substitution therapy, was effective, and supports recommendations within the UK, Europe, and globally on the need to expand NSP and opiate substitution therapy to prevent HCV infection. The question remains on what levels of opiate substitution therapy and NSP coverage (and behaviour change) are required to drive down HCV prevalence (and whether these are sustainable). |
| Wright and Tompkins **(**Leeds Community Drug Treatment Services and North East Leeds Primary Care Trust) | How can health services effectively meet the health needs of homeless people? | 2006 | Undertaken in the UK, focus was international. | To critically examine the international literature pertaining to the healthcare of homeless people and discuss the effectiveness of treatment interventions. | Electronic databases were reviewed using key terms relating to homelessness, intervention studies, drug misuse, alcohol misuse, and mental health. The review was not limited to publications in English. Grey literature search and discussion with experts were conducted. Literature from 1966 to 2003 included. | Not reported. | Not specified – potentially any study type. | Interventions such as: primary prevention interventions; management of drug dependence; medically supervised injecting centres; sexual health promotion; and management of alcohol dependence. | All drugs (including alcohol). | Looked at people who are homeless but, in their search, included key terms relating to substance use, alcohol use, and mental health. | Effective interventions for drug dependence include adequate oral opiate maintenance therapy, tetanus and hepatitis A and B immunisation, safer injecting advice, and access to needle exchange programmes. There is emerging evidence for the effectiveness of supervised injecting rooms for homeless injecting drug users and for the peer distribution of take-home naloxone in reducing drug-related deaths. There is some evidence that assertive outreach programmes for those with mental ill health, supportive programmes to aid those with the motivation to address alcohol dependence, and informal interactive programmes to promote sexual health can lead to lasting health gain. | Effective interventions for drug dependence include adequate oral opiate maintenance therapy, tetanus and hepatitis A and B immunisation, safer injecting advice, and access to needle exchange programmes. Emerging evidence for the effectiveness of supervised injecting rooms and for the peer distribution of take home naloxone (THN) in reducing drug-related deaths. Some evidence that assertive outreach programmes for those with mental ill health, supportive programmes to help address alcohol dependence, and informal programmes to promote sexual health can lead to lasting health gain. | As multiple morbidity is common among homeless people, accessible and available primary healthcare is a prerequisite for effective health interventions. This requires addressing barriers to provision and multi-agency working so that homeless people can access the full range of health and social care services. There are examples of best practice in the treatment and retention of homeless people in health and social care, and such models can inform future provision. |
| Wright and Walker (Leeds Community Drug Treatment Services, Centre for Research in Primary Care, UK, and School of Health and Community Care, Leeds Metropolitan University, UK) | Homelessness and drug use – a narrative systematic review of interventions to promote sexual health. | 2006 | Undertaken in the UK, focus was international but only “high-income countries”. | The objective of this research project was to examine the effectiveness of sexual health promotion interventions in homeless drug-using populations. | Electronic databases (1966 to 2003) were searched. Two independent researchers selected studies for inclusion.  Relevant journals from the preceding five years and reference lists were also screened for additional studies. There were no language restrictions and unpublished literature was sought from experts in the field. | Six studies included. | All longitudinal controlled study designs, including RCTs, quasi-experimental, and non-experimental designs, were eligible. Studies that evaluated interventions to promote sexual health were eligible for inclusion. | A significant proportion of the included studies compared ‘traditional’ and ‘specialised’ multicomponent AIDS-focused programmes that involved group educational and practical elements. Other studies included counselling, and benefits/housing assistance. The study reviewed interventions which targeted the individual. Such interventions attempt to modify the knowledge, attitudes, motives, skills, and/or physiological state of individuals in the target population. | All drugs (including alcohol). | Study selection was restricted to homeless populations residing in high-income, ‘developed’ countries. It excluded backpacker populations and asylum-seeking populations from the review. | Comparison of ‘traditional’ and ‘specialised’ multicomponent AIDS-focused programmes that involved group educational and practical elements. Other studies included counselling, and benefits/housing assistance. Programme offered HIV education; alcohol and drug counselling; benefits and housing assistance; ‘traditional’ intervention of AIDS videotapes and 1-hour group session (covering AIDS education, HIV testing, condom use, use of bleach to sterilise injecting equipment, and a list of community resources). ‘Specialised’ intervention of AIDS videotape and 2-hour session tailored to individual learning needs. This included demonstration and return demonstration of risk-reducing behaviours, discussion of coping skills, and enhancing self-esteem. | There is concordance between the studies that sexual health promotion interventions resulted in increased knowledge of both sexual risk and drug issues. The interventions also initially led to a reduction in sexual risk and a reduction in drug use. There was not concordance between the studies regarding whether the effect of the intervention was sustained over a 2-year period. The relationship between the intervention and psychosocial outcomes appeared to be more complex. Broadly speaking, the interventions improved psychosocial functioning. | Brief interventions appear to confer significant benefit. However, there is a need for further implementation and evaluation of health promotion activities in the UK context which target the individual, the social setting, and societal structure. There is a need to formally plan, initiate, and evaluate interventions to promote sexual health among UK homeless drug-using populations. |
