# Supplementary material for: What treatment and services are effective for people who are homeless and use drugs? A systematic ‘review of reviews’
Source: PLoS One. 2021 Jul 14;16(7):e0254729. doi: 10.1371/journal.pone.0254729 (PMC8279330; doi:10.1371/journal.pone.0254729)
Supplement: S1 Data — (DOCX) [file pone.0254729.s005.docx]

**S1 Data. Search strategy**

**MEDLINE (Ovid – All MEDLINE)**

1 exp Homeless Persons/

2 homeless*.ti,ab,kw.

3 undomiciled.ti,ab,kw.

4 houseless*.ti,ab,kw.

5 (rough adj sleep*).ti,ab,kw.

6 "street person".ti,ab,kw.

7 "street people".ti,ab,kw.

8 vagrant*.ti,ab,kw.

9 "no fixed abode".ti,ab,kw.

10 (transient adj3 (people or person* or adult* or man or men or woman or women

or individual* or population* or group* or communit*)).ti,ab,kw.

11 (shelter adj (seek* or using or need*)).ti,ab,kw.

12 "shelter use".ti,ab,kw.

13 "unstabl* hous*".ti,ab,kw.

14 unshelter*.ti,ab,kw.

15 ((emergency or temporary or overnight or housed or intermittent or night) adj

(shelter* or hostel*)).ti,ab,kw.

16 roofless*.ti,ab,kw.

17 destitute.ti,ab,kw.

18 runaway*.ti,ab,kw.

19 ((without or lack) adj2 (home* or housing or house)).ti,ab,kw.

20 or/1-19

21 ((Drug* or substance* or polydrug or "poly-drug" or "legal high*" or

psychoactive* or "psycho-active*" or psychotropic*) adj4 (use* or abus* or misuse* or

"mis-use*" or refus* or problem* or taking or take* or experiment* or

addict*)).ti,ab,kw.

22 ((ketamine or speed or spice or cocaine or crack or mushroom* or solvent* or

inhalant or "nitrous oxide" or "laughing gas" or benzodiazepine* or tranquiliser* or

tranquilizer* or opioid* or opiate*or hallucinogen* or "anabolic steroid*" or gabapentin

or pregabalin or etizolam or valium) adj4 (use* or abus* or misuse* or "mis-use*" or

refus* or problem* or taking or take* or experiment* or addict*)).ti,ab,kw.

23 (Cannab* or marijuana or skunk or ecstasy or MDMA or LSD or "lysergic acid

diethylamide" or amphetamine* or amfetamin* or mephedrone or mkat or "meow

meow" or meth or methamphetamine or methamfetamin* or psychedelic* or pcp or

phencyclidine or "anabolic steroid*" or ped or peds or pied or pieds or "performance

enhancing" or "image enhancing" or heroin or poppers or "amyl nitrate" or "butyl

nitrate" or "new psychoactive drug*" or "novel psychoactive drug*" or "novel

psychoactive substance*" or NPS or "harm reduction" or detox*).ti,ab,kw.

24 exp Street Drugs/

25 exp Designer Drugs/

26 exp Marijuana Abuse/

27 exp Drug-Seeking Behavior/

28 exp Performance-Enhancing Substances/

29 exp Substance-Related Disorders/

30 exp Amphetamine-Related Disorders/

31 exp Cocaine-Related Disorders/

32 exp Inhalant Abuse/

33 exp Marijuana Abuse/

34 exp Opioid-Related Disorders/

35 exp Phencyclidine Abuse/

36 exp Substance Abuse, Intravenous/

37 exp Marijuana Smoking/

38 exp Drug Users/

39 or/21-38

40 20 and 39

41 limit 40 to (meta analysis or "systematic review")

42 "systematic review".ti,ab,kw.

43 "evidence synthesis".ti,ab,kw.

44 "realist review".ti,ab,kw.

45 "realist synthesis".ti,ab,kw.

46 "meta analysis".ti,ab,kw.

47 "mixed methods review".ti,ab,kw.

48 "meta-synthesis".ti,ab,kw.

49 "mixed methods synthesis".ti,ab,kw.

50 meta-epidemiology.ti,ab,kw.

51 "qualitative synthesis".ti,ab,kw.

52 ("meta-epidemiolog*" or meta-ethnograph*).ti,ab,kw.

53 "cochrane review".ti,ab,kw.

54 "integrative review".ti,ab,kw.

55 "umbrella review".ti,ab,kw.

56 "critical interpretive synthesis".ti,ab,kw.

57 or/42-56

58 40 and 57

59 41 or 58

60 from 59 keep 1-101

**CINAHL (EBSCOHOST)**

| S1 | (MH "Homeless Persons") OR (MH "Homelessness") |  |
| --- | --- | --- |
| S2 | TI homeless* OR AB homeless* OR SU homeless* |  |
| S3 | TI undomiciled OR AB undomiciled OR SU undomiciled |  |
| S4 | TI houseless* OR AB houseless* OR SU houseless* |  |
| S5 | TI (rough NEAR sleep*) OR AB (rough NEAR sleep*) OR SU (rough NEAR sleep*) |  |
| S6 | TI "street person" OR AB "street person" OR SU "street person" |  |
| S7 | TI "street people" OR AB "street people" OR SU "street people" |  |
| S8 | TI vagrant* OR AB vagrant* OR SU vagrant* |  |
| S9 | TI "no fixed abode" OR AB "no fixed abode" OR SU "no fixed abode" |  |
| S10 | TI ( (transient N3 (people or person* or adult* or man or men or woman or women or individual* or population* or group* or communit*)) ) OR AB ( (transient N3 (people or person* or adult* or man or men or woman or women or individual* or population* or group* or communit*)) ) OR SU ( (transient N3 (people or person* or adult* or man or men or woman or women or individual* or population* or group* or communit*)) ) |  |
| S11 | TI ( (shelter N (seek* or using or need*)) ) OR AB ( (shelter N (seek* or using or need*)) ) OR SU ( (shelter N (seek* or using or need*)) ) |  |
| S12 | TI "shelter use" OR AB "shelter use" OR SU "shelter use" |  |
| S13 | TI "unstabl* hous*" OR AB "unstabl* hous*" OR SU "unstabl* hous*" |  |
| S14 | TI unshelter* OR AB unshelter* OR SU unshelter* |  |
| S15 | TI ( ((emergency or temporary or overnight or housed or intermittent or night) N (shelter* or hostel*)) ) OR AB ( ((emergency or temporary or overnight or housed or intermittent or night) N (shelter* or hostel*)) ) OR SU ( ((emergency or temporary or overnight or housed or intermittent or night) N (shelter* or hostel*)) ) |  |
| S16 | TI roofless* OR AB roofless* OR SU roofless* |  |
| S17 | TI destitute OR AB destitute OR SU destitute |  |
| S18 | TI runaway* OR AB runaway* OR SU runaway* |  |
| S19 | TI ( ((without or lack) N2 (home* or housing or house)) ) OR AB ( ((without or lack) N2 (home* or housing or house)) ) OR SU ( ((without or lack) N2 (home* or housing or house)) ) |  |
| S20 | S1 OR S2 OR S3 OR S4 OR S5 OR S6 OR S7 OR S8 OR S9 OR S10 OR S11 OR S12 OR S13 OR S14 OR S15 OR S16 OR S17 OR S18 OR S19 |  |
| S21 | TI ( ((Drug* or substance* or polydrug or "poly-drug" or "legal high*" or psychoactive* or "psycho-active*" or psychotropic*) N4 (use* or abus* or misuse* or "mis-use*" or refus* or problem* or taking or take* or experiment* or addict*)) ) OR AB ( ((Drug* or substance* or polydrug or "poly-drug" or "legal high*" or psychoactive* or "psycho-active*" or psychotropic*) N4 (use* or abus* or misuse* or "mis-use*" or refus* or problem* or taking or take* or experiment* or addict*)) ) OR SU ( ((Drug* o [...](javascript:showHistoryTerm('ctl00_ctl00_FindField_FindField_historyControl_HistoryRepeater_ctl20_ellipsis',true)) |  |
| S22 | TI ( ((ketamine or speed or spice or cocaine or crack or mushroom* or solvent* or inhalant or "nitrous oxide" or "laughing gas" or benzodiazepine* or tranquiliser* or tranquilizer* or opioid* or opiate*or hallucinogen* or "anabolic steroid*" or gabapentin or pregabalin or etizolam or valium) N4 (use* or abus* or misuse* or "mis-use*" or refus* or problem* or taking or take* or experiment* or addict*)) ) OR AB ( ((ketamine or speed or spice or cocaine or crack or mushroom* or solvent* or inhalant [...](javascript:showHistoryTerm('ctl00_ctl00_FindField_FindField_historyControl_HistoryRepeater_ctl21_ellipsis',true)) |  |
| S23 | TI ( (Cannab* or marijuana or skunk or ecstasy or MDMA or LSD or "lysergic acid diethylamide" or amphetamine* or amfetamin* or mephedrone or mkat or "meow meow" or meth or methamphetamine or methamfetamin* or psychedelic* or pcp or phencyclidine or "anabolic steroid*" or ped or peds or pied or pieds or "performance enhancing" or "image enhancing" or heroin or poppers or "amyl nitrate" or "butyl nitrate" or "new psychoactive drug*" or "novel psychoactive drug*" or "novel psychoactive substance*"  [...](javascript:showHistoryTerm('ctl00_ctl00_FindField_FindField_historyControl_HistoryRepeater_ctl22_ellipsis',true)) |  |
| S24 | (MH "Street Drugs+") |  |
| S25 | (MH "Designer Drugs") |  |
| S26 | (MH "Substance Abusers+") |  |
| S27 | (MH "Drug-Seeking Behavior") |  |
| S28 | (MH "Substance Use Disorders+") |  |
| S29 | (MH "Inhalant Abuse") |  |
| S30 | (MH "Substance Dependence+") |  |
| S31 | (TI ("harm reduction" or detox*)) OR (AB ("harm reduction" or detox*)) OR (SU ("harm reduction" or detox*)) |  |
| S32 | S21 OR S22 OR S23 OR S24 OR S25 OR S26 OR S27 OR S28 OR S29 OR S30 OR S31 |  |
| S33 | S20 AND S32 |  |
| S34 | TI "systematic review" OR AB "systematic review" OR SU "systematic review" |  |
| S35 | TI "evidence synthesis" OR AB "evidence synthesis" OR SU "evidence synthesis" |  |
| S36 | TI "realist review" OR AB "realist review" OR SU "realist review" |  |
| S37 | TI "realist synthesis" OR AB "realist synthesis" OR SU "realist synthesis" |  |
| S38 | TI "meta analysis" OR AB "meta analysis" OR SU "meta analysis" |  |
| S39 | TI "mixed methods review" OR AB "mixed methods review" OR SU "mixed methods review" |  |
| S40 | TI "meta-synthesis" OR AB "meta-synthesis" OR SU "meta-synthesis" |  |
| S41 | TI "mixed methods synthesis" OR AB "mixed methods synthesis" OR SU "mixed methods synthesis" |  |
| S42 | TI "qualitative synthesis" OR AB "qualitative synthesis" OR SU "qualitative synthesis" |  |
| S43 | (TI ("meta-epidemiolog*" or "meta-ethnograph*")) OR (AB ("meta-epidemiolog*" or "meta-ethnograph*")) OR (SU ("meta-epidemiolog*" or "meta-ethnograph*")) |  |
| S44 | TI "cochrane review" OR AB "cochrane review" OR SU "cochrane review" |  |
| S45 | TI "integrative review" OR AB "integrative review" OR SU "integrative review" |  |
| S46 | TI "umbrella review" OR AB "umbrella review" OR SU "umbrella review" |  |
| S47 | TI "critical interpretive synthesis" OR AB "critical interpretive synthesis" OR SU "critical interpretive synthesis" |  |
| S48 | S34 OR S35 OR S36 OR S37 OR S38 OR S39 OR S40 OR S41 OR S42 OR S43 OR S44 OR S45 OR S46 OR S47 |  |
| S49 | S33 AND S48 |  |
| S50 | S20 AND S32 |  |
| S51 | S49 OR S50 |  |

**EMBASE (OVID)**

1. exp homelessness/ or exp homeless person/

2. homeless*.ti,ab,kw.

3. undomiciled.ti,ab,kw.

4. houseless*.ti,ab,kw.

5. (rough adj sleep*).ti,ab,kw.

6. "street person".ti,ab,kw.

7. "street people".ti,ab,kw.

8. vagrant*.ti,ab,kw.

9. "no fixed abode".ti,ab,kw.

10. (transient adj3 (people or person* or adult* or man or men or woman or women or individual* or population* or group* or communit*)).ti,ab,kw.

11. (shelter adj (seek* or using or need*)).ti,ab,kw.

12. "shelter use".ti,ab,kw.

13. "unstabl* hous*".ti,ab,kw.

14. unshelter*.ti,ab,kw.

15. ((emergency or temporary or overnight or housed or intermittent or night) adj (shelter* or hostel*)).ti,ab,kw.

16. roofless*.ti,ab,kw.

17. destitute.ti,ab,kw.

18. runaway*.ti,ab,kw.

19. ((without or lack) adj2 (home* or housing or house)).ti,ab,kw.

20. or/1-19

21. ((Drug* or substance* or polydrug or "poly-drug" or "legal high*" or psychoactive* or "psycho-active*" or psychotropic*) adj4 (use* or abus* or misuse* or "mis-use*" or refus* or problem* or taking or take* or experiment* or addict*)).ti,ab,kw.

22. ((ketamine or speed or spice or cocaine or crack or mushroom* or solvent* or inhalant or "nitrous oxide" or "laughing gas" or benzodiazepine* or tranquiliser* or tranquilizer* or opioid* or opiate*or hallucinogen* or "anabolic steroid*" or gabapentin or pregabalin or etizolam or valium) adj4 (use* or abus* or misuse* or "mis-use*" or refus* or problem* or taking or take* or experiment* or addict*)).ti,ab,kw.

23. (Cannab* or marijuana or skunk or ecstasy or MDMA or LSD or "lysergic acid diethylamide" or amphetamine* or amfetamin* or mephedrone or mkat or "meow meow" or meth or methamphetamine or methamfetamin* or psychedelic* or pcp or phencyclidine or "anabolic steroid*" or ped or peds or pied or pieds or "performance enhancing" or "image enhancing" or heroin or poppers or "amyl nitrate" or "butyl nitrate" or "new psychoactive drug*" or "novel psychoactive drug*" or "novel psychoactive substance*" or NPS or "harm reduction" or detox*).ti,ab,kw.

24. exp street drug/

25. exp designer drug/

26. exp cannabis addiction/

27. exp drug seeking behavior/

28. exp performance enhancing substance/

29. exp drug dependence/

30. exp amphetamine dependence/

31. exp cocaine dependence/

32. exp inhalant abuse/

33. exp opiate addiction/

34. exp phencyclidine abuse/

35. exp substance abuse/

36. exp cannabis smoking/

37. exp drug abuse/

38. or/21-37

39. 20 and 38

40. limit 39 to (meta analysis or "systematic review")

41. "systematic review".ti,ab,kw.

42. "evidence synthesis".ti,ab,kw.

43. "realist review".ti,ab,kw.

44. "realist synthesis".ti,ab,kw.

45. "meta analysis".ti,ab,kw.

46. "mixed methods review".ti,ab,kw.

47. "meta-synthesis".ti,ab,kw.

48. "mixed methods synthesis".ti,ab,kw.

49. "qualitative synthesis".ti,ab,kw.

50. ("meta-epidemiolog*" or meta-ethnograph*).ti,ab,kw.

51. "cochrane review".ti,ab,kw.

52. "integrative review".ti,ab,kw.

53. "umbrella review".ti,ab,kw.

54. "critical interpretive synthesis".ti,ab,kw.

55. or/41-54

56. 39 and 55

57. 40 or 56

**PSYCHINFO (OCID)**

1. exp Homeless/

2. homeless*.ti,ab,id.

3. undomiciled.ti,ab,id.

4. houseless*.ti,ab,id.

5. (rough adj sleep*).ti,ab,id.

6. "street person".ti,ab,id.

7. "street people".ti,ab,id.

8. vagrant*.ti,ab,id.

9. "no fixed abode".ti,ab,id.

10. (transient adj3 (people or person* or adult* or man or men or woman or women or individual* or population* or group* or communit*)).ti,ab,id.

11. (shelter adj (seek* or using or need*)).ti,ab,id.

12. "shelter use".ti,ab,id.

13. "unstabl* hous*".ti,ab,id.

14. unshelter*.ti,ab,id.

15. ((emergency or temporary or overnight or housed or intermittent or night) adj (shelter* or hostel*)).ti,ab,id.

16. roofless*.ti,ab,id.

17. destitute.ti,ab,id.

18. runaway*.ti,ab,id.

19. ((without or lack) adj2 (home* or housing or house)).ti,ab,id.

20. 1 or 2 or 3 or 4 or 5 or 6 or 7 or 8 or 9 or 10 or 11 or 12 or 13 or 14 or 15 or 16 or 17 or 18 or 19

21. ((Drug* or substance* or polydrug or "poly-drug" or "legal high*" or psychoactive* or "psycho-active*" or psychotropic*) adj4 (use* or abus* or misuse* or "mis-use*" or refus* or problem* or taking or take* or experiment* or addict*)).ti,ab,id.

22. ((ketamine or speed or spice or cocaine or crack or mushroom* or solvent* or inhalant or "nitrous oxide" or "laughing gas" or benzodiazepine* or tranquiliser* or tranquilizer* or opioid* or opiate*or hallucinogen* or "anabolic steroid*" or gabapentin or pregabalin or etizolam or valium) adj4 (use* or abus* or misuse* or "mis-use*" or refus* or problem* or taking or take* or experiment* or addict*)).ti,ab,id.

23. (Cannab* or marijuana or skunk or ecstasy or MDMA or LSD or "lysergic acid diethylamide" or amphetamine* or amfetamin* or mephedrone or mkat or "meow meow" or meth or methamphetamine or methamfetamin* or psychedelic* or pcp or phencyclidine or "anabolic steroid*" or ped or peds or pied or pieds or "performance enhancing" or "image enhancing" or heroin or poppers or "amyl nitrate" or "butyl nitrate" or "new psychoactive drug*" or "novel psychoactive drug*" or "novel psychoactive substance*" or NPS or "harm reduction" or detox*).ti,ab,id.

24. exp Drug Abuse/

25. exp Designer Drugs/

26. exp Drug Dependency/

27. exp Marijuana Usage/

28. exp Drug Seeking/

29. exp Drug Addiction/

30. exp Performance Enhancing Drugs/

31. exp "Substance Use Disorder"/

32. exp Inhalant Abuse/

33. exp "Substance Use Treatment"/

34. or/21-33

35. 20 and 34

36. "systematic review".ti,ab,id.

37. "evidence synthesis".ti,ab,id.

38. "realist review".ti,ab,id.

39. "realist synthesis".ti,ab,id.

40. "meta analysis".ti,ab,id.

41. "mixed methods review".ti,ab,id.

42. "meta-synthesis".ti,ab,id.

43. "meta-synthesis".ti,ab,id.

44. "qualitative synthesis".ti,ab,id.

45. ("meta-epidemiolog*" or meta-ethnograph*).ti,ab,id.

46. "cochrane review".ti,ab,id.

47. "integrative review".ti,ab,id.

48. "umbrella review".ti,ab,id.

49. "critical interpretive synthesis".ti,ab,id.

50. 36 or 37 or 38 or 39 or 40 or 41 or 42 or 43 or 44 or 45 or 46 or 47 or 48 or 49

51. 35 and 50

**Cochrane Database of Systematic Reviews (The Cochrane Library)**

#1 MeSH descriptor: [Homeless Persons] explode all trees

#2 homeless*:ti,ab

#3 undomiciled:ti,ab

#4 houseless*:ti,ab

#5 (rough NEAR sleep*):ti,ab

#6 "street person":ti,ab

#7 "street people":ti,ab

#8 vagrant*:ti,ab

#9 "no fixed abode":ti,ab

#10 (transient NEAR/3 (people or person* or adult* or man or men or woman or women

or individual* or population* or group* or communit*)):ti,ab

#11 (shelter NEAR (seek* or using or need*))

#12 "shelter use":ti,ab

#13 "unstabl* hous*":ti,ab

#14 unshelter*:ti,ab

#15 ((emergency or temporary or overnight or housed or intermittent or night) NEAR

(shelter* or hostel*)):ti,ab

#16 roofless*:ti,ab

#17 destitute:ti,ab

#18 runaway*:ti,ab

#19 ((without or lack) NEAR/2 (home* or housing or house)):ti,ab

#20 {OR #1-#19}

#21 ((Drug* or substance* or polydrug or "poly-drug" or "legal high*" or

psychoactive* or "psycho-active*" or psychotropic*) NEAR/4 (use* or abus* or misuse* or

"mis-use*" or refus* or problem* or taking or take* or experiment* or

addict*)):ti,ab

#22 ((ketamine or speed or spice or cocaine or crack or mushroom* or solvent* or

inhalant or "nitrous oxide" or "laughing gas" or benzodiazepine* or tranquiliser* or

tranquilizer* or opioid* or opiate*or hallucinogen* or "anabolic steroid*" or gabapentin

or pregabalin or etizolam or valium) NEAR/4 (use* or abus* or misuse* or "mis-use*" or

refus* or problem* or taking or take* or experiment* or addict*)):ti,ab

#23 (Cannab* or marijuana or skunk or ecstasy or MDMA or LSD or "lysergic acid

diethylamide" or amphetamine* or amfetamin* or mephedrone or mkat or "meow

meow" or meth or methamphetamine or methamfetamin* or psychedelic* or pcp or

phencyclidine or "anabolic steroid*" or ped or peds or pied or pieds or "performance

enhancing" or "image enhancing" or heroin or poppers or "amyl nitrate" or "butyl

nitrate" or "new psychoactive drug*" or "novel psychoactive drug*" or "novel

psychoactive substance*" or NPS or "harm reduction" or detox*):ti,ab

#24 MeSH descriptor: [Street Drugs] explode all trees

#25 MeSH descriptor: [Designer Drugs] explode all trees

#26 MeSH descriptor: [Marijuana Abuse] explode all trees

#27 MeSH descriptor: [Drug-Seeking Behavior] explode all trees

#28 MeSH descriptor: [Performance-Enhancing Substances] explode all trees

#29 MeSH descriptor: [Substance-Related Disorders] explode all trees

#30 MeSH descriptor: [Amphetamine-Related Disorders] explode all trees

#31 MeSH descriptor: [Cocaine-Related Disorders] explode all trees

#32 MeSH descriptor: [Inhalant Abuse] explode all trees

#33 MeSH descriptor: [Marijuana Abuse] explode all trees

#34 MeSH descriptor: [Opioid-Related Disorders] explode all trees

#35 MeSH descriptor: [Phencyclidine Abuse] explode all trees

#36 MeSH descriptor: [Substance-Related Disorders] explode all trees

#37 MeSH descriptor: [Marijuana Smoking] explode all trees

#38 MeSH descriptor: [Drug Users] explode all trees

#39 {OR #21-#38}

#40 #20 AND #39

**PROSPERO**

#1 MeSH DESCRIPTOR Homeless Persons EXPLODE ALL TREES

#2 homeless* or undomiciled or houseless* or "rough sleep*" or shelter or "street person" or "street people" or vagrant* or "no fixed abode" or shelter or "unstabl* hous*" or hostel* or unshelter* or roofless* or destitute or runaway* or "without home*" or "without housing" or "without house" or "lack home*" or "lack housing" or "lack house"

#3 #1 OR #2

#4 Drug* or substance* or polydrug or "poly-drug" or "legal high*" or psychoactive* or "psycho-active*" or psychotropic* or ketamine or speed or spice or cocaine or crack or mushroom* or solvent* or inhalant or "nitrous oxide" or "laughing gas" or benzodiazepine* or tranquiliser* or tranquilizer* or opioid* or opiate*or hallucinogen* or "anabolic steroid*" or gabapentin or pregabalin or etizolam or valium or Cannab* or marijuana or skunk or ecstasy or MDMA or LSD or "lysergic acid diethylamide" or amphetamine* or amfetamin* or mephedrone or mkat or "meow meow" or meth or methamphetamine or methamfetamin* or psychedelic* or pcp or phencyclidine or "anabolic steroid*" or ped or peds or pied or pieds or "performance enhancing" or "image enhancing" or heroin or poppers or "amyl nitrate" or "butyl nitrate" or "new psychoactive drug*" or "novel psychoactive drug*" or "novel psychoactive substance*" or NPS or "harm reduction" or detox*

#5 MeSH DESCRIPTOR Street Drugs EXPLODE ALL TREES

#6 MeSH DESCRIPTOR Designer Drugs EXPLODE ALL TREES

#7 MeSH DESCRIPTOR Marijuana Abuse EXPLODE ALL TREES

#8 MeSH DESCRIPTOR Drug-Seeking Behavior EXPLODE ALL TREES

#9 MeSH DESCRIPTOR Performance-Enhancing Substances EXPLODE ALL TREES

#10 MeSH DESCRIPTOR Substance-Related Disorders EXPLODE ALL TREES

#11 MeSH DESCRIPTOR Amphetamine-Related Disorders EXPLODE ALL TREES

#12 MeSH DESCRIPTOR Cocaine-Related Disorders EXPLODE ALL TREES

#13 MeSH DESCRIPTOR Inhalant Abuse EXPLODE ALL TREES

#14 MeSH DESCRIPTOR Marijuana Abuse EXPLODE ALL TREES

#15 MeSH DESCRIPTOR Opioid-Related Disorders EXPLODE ALL TREES

#16 MeSH DESCRIPTOR Phencyclidine Abuse EXPLODE ALL TREES

#17 MeSH DESCRIPTOR Substance Abuse, Intravenous EXPLODE ALL TREES

#18 MeSH DESCRIPTOR Marijuana Smoking EXPLODE ALL TREES

#19 MeSH DESCRIPTOR Drug Users EXPLODE ALL TREES

#20 #19 OR #18 OR #17 OR #16 OR #15 OR #14 OR #13 OR #12 OR #11 OR #10 OR #8 OR #9 OR #7 OR #6 OR #5 OR #4

#21 #3 AND #20

**Epistemonikos**

(homeless* OR undomiciled OR houseless* OR "rough sleep*" OR shelter OR "street person" OR "street people" OR vagrant* OR "no fixed abode" OR transient OR "unstabl* hous*" OR hostel* OR unshelter* OR roofless* OR destitute OR runaway* OR "without home*" OR "without housing" OR "without house" OR "lack home*" OR "lack housing" OR "lack house") AND (Drug* OR substance* OR polydrug OR "poly-drug" OR "legal high*" OR psychoactive* OR "psycho-active*" OR psychotropic* OR ketamine OR speed OR spice OR cocaine OR crack OR mushroom* OR solvent* OR inhalant OR "nitrous oxide" OR "laughing gas" OR benzodiazepine* OR tranquiliser* OR tranquilizer* OR opioid* OR opiate*or hallucinogen* OR "anabolic steroid*" OR gabapentin OR pregabalin OR etizolam OR valium OR Cannab* OR marijuana OR skunk OR ecstasy OR MDMA OR LSD OR "lysergic acid diethylamide" OR amphetamine* OR amfetamin* OR mephedrone OR mkat OR "meow meow" OR meth OR methamphetamine OR methamfetamin* OR psychedelic* OR pcp OR phencyclidine OR "anabolic steroid*" OR ped OR peds OR pied OR pieds OR "performance enhancing" OR "image enhancing" OR heroin OR poppers OR "amyl nitrate" OR "butyl nitrate" OR "new psychoactive drug*" OR "novel psychoactive drug*" OR "novel psychoactive substance*" OR NPS OR "harm reduction" OR detox* OR "drug* use*" OR "drug* abuse*" OR "drug* misuse" OR "drug* mis-use*" OR "drug* addict*" OR "drug* taker" OR "drug taking" OR "drug* problem*" OR "substance* use*" OR "substance* abuse*" OR "substance* misuse" OR "substance* mis-use*" OR "substance* addict*" OR "substance* problem*" OR "substance* taker" OR "substance taking" OR polydrug OR "poly-drug" OR "legal high*" OR psychoactive* OR "psycho-active*" OR psychotropic*)

**The Campbell Collaboration**

| **Search term** | **Results** |
| --- | --- |
| [homeless](https://www.campbellcollaboration.org/component/jak2filter/?Itemid=1352&issearch=1&isc=1&category_id=101&searchword=homeless&xf_8%5b0%5d=3&ordering=publishUp)/homelessness^1^ | 3 |
| [undomiciled](https://www.campbellcollaboration.org/component/jak2filter/?Itemid=1352&issearch=1&isc=1&category_id=101&searchword=homeless&xf_8%5b0%5d=3&ordering=publishUp) | 0 |
| [houseless](https://www.campbellcollaboration.org/component/jak2filter/?Itemid=1352&issearch=1&isc=1&category_id=101&searchword=homeless&xf_8%5b0%5d=3&ordering=publishUp) | 0 |
| [“rough sleep*”](https://www.campbellcollaboration.org/component/jak2filter/?Itemid=1352&issearch=1&isc=1&category_id=101&searchword=homeless&xf_8%5b0%5d=3&ordering=publishUp) | 0 |
| [“street person”](https://www.campbellcollaboration.org/component/jak2filter/?Itemid=1352&issearch=1&isc=1&category_id=101&searchword=homeless&xf_8%5b0%5d=3&ordering=publishUp) | 0 |
| [“street people”](https://www.campbellcollaboration.org/component/jak2filter/?Itemid=1352&issearch=1&isc=1&category_id=101&searchword=homeless&xf_8%5b0%5d=3&ordering=publishUp) | 0 |
| [shelter](https://www.campbellcollaboration.org/component/jak2filter/?Itemid=1352&issearch=1&isc=1&category_id=101&searchword=homeless&xf_8%5b0%5d=3&ordering=publishUp) | 2 |
| [vagrant*](https://www.campbellcollaboration.org/component/jak2filter/?Itemid=1352&issearch=1&isc=1&category_id=101&searchword=homeless&xf_8%5b0%5d=3&ordering=publishUp) | 0 |
| [“no fixed abode”](https://www.campbellcollaboration.org/component/jak2filter/?Itemid=1352&issearch=1&isc=1&category_id=101&searchword=homeless&xf_8%5b0%5d=3&ordering=publishUp) | 0 |
| [transient](https://www.campbellcollaboration.org/component/jak2filter/?Itemid=1352&issearch=1&isc=1&category_id=101&searchword=homeless&xf_8%5b0%5d=3&ordering=publishUp) | 0 |
| ["unstable housing"](https://www.campbellcollaboration.org/component/jak2filter/?Itemid=1352&issearch=1&isc=1&category_id=101&searchword=homeless&xf_8%5b0%5d=3&ordering=publishUp) | 0 |
| [hostel](https://www.campbellcollaboration.org/component/jak2filter/?Itemid=1352&issearch=1&isc=1&category_id=101&searchword=homeless&xf_8%5b0%5d=3&ordering=publishUp) | 0 |
| [unshelter](https://www.campbellcollaboration.org/component/jak2filter/?Itemid=1352&issearch=1&isc=1&category_id=101&searchword=homeless&xf_8%5b0%5d=3&ordering=publishUp) | 0 |
| [roofless](https://www.campbellcollaboration.org/component/jak2filter/?Itemid=1352&issearch=1&isc=1&category_id=101&searchword=homeless&xf_8%5b0%5d=3&ordering=publishUp) | 0 |
| [destitute](https://www.campbellcollaboration.org/component/jak2filter/?Itemid=1352&issearch=1&isc=1&category_id=101&searchword=homeless&xf_8%5b0%5d=3&ordering=publishUp) | 0 |
| [runaway*](https://www.campbellcollaboration.org/component/jak2filter/?Itemid=1352&issearch=1&isc=1&category_id=101&searchword=homeless&xf_8%5b0%5d=3&ordering=publishUp) | 0 |
| [“without home”](https://www.campbellcollaboration.org/component/jak2filter/?Itemid=1352&issearch=1&isc=1&category_id=101&searchword=homeless&xf_8%5b0%5d=3&ordering=publishUp) | 0 |
| ["without housing"](https://www.campbellcollaboration.org/component/jak2filter/?Itemid=1352&issearch=1&isc=1&category_id=101&searchword=homeless&xf_8%5b0%5d=3&ordering=publishUp) | 0 |
| ["without house"](https://www.campbellcollaboration.org/component/jak2filter/?Itemid=1352&issearch=1&isc=1&category_id=101&ordering=publishUp) | 0 |
| ["lack home"](file:///C:\Users\Michelle\Documents\Independent%20Training\Harry%20LJMU\campbellcollaboration.org\component\jak2filter\%3fItemid=1352&issearch=1&isc=1&category_id=101&ordering=publishUp) | 0 |
| ["lack housing"](https://www.campbellcollaboration.org/component/jak2filter/?Itemid=1352&issearch=1&isc=1&category_id=101&ordering=publishUp) | 0 |
| ["lack house"](https://www.campbellcollaboration.org/component/jak2filter/?Itemid=1352&issearch=1&isc=1&category_id=101&ordering=publishUp) | 0 |

Notes

1. Homeless also retrieves records with homelessness
2. Cannot search using truncation *
3. Does not like long strings of search terms

**NIHR Journals Library (HTA)**

| **Search term** |
| --- |
| [Homeless*](https://www.journalslibrary.nihr.ac.uk/search/#/?search=homeless*&tab=All&page=1&rows=25&orderby=score&publicationrange=publicationdate:%5B*%20TO%20*%5D&chiefinvestigator=&doi=&projectreference=&title=&author=&leadauthor=&direction=desc&selected_facets=researchtype:%22Evidence%20Synthesis%22) |
| [undomiciled](https://www.journalslibrary.nihr.ac.uk/search/#/?search=undomiciled&tab=All&page=1&rows=25&orderby=score&publicationrange=publicationdate:%5B*%20TO%20*%5D&chiefinvestigator=&doi=&projectreference=&title=&author=&leadauthor=&direction=desc&selected_facets=researchtype:%22Evidence%20Synthesis%22) |
| [Houseless*](https://www.journalslibrary.nihr.ac.uk/search/#/?search=houseless*&tab=All&page=1&rows=25&orderby=score&publicationrange=publicationdate:%5B*%20TO%20*%5D&chiefinvestigator=&doi=&projectreference=&title=&author=&leadauthor=&direction=desc&selected_facets=researchtype:%22Evidence%20Synthesis%22) |
| [“rough sleep*”](https://www.journalslibrary.nihr.ac.uk/search/#/?search=%22rough%20sleep*%22&tab=All&page=1&rows=25&orderby=score&publicationrange=publicationdate:%5B*%20TO%20*%5D&chiefinvestigator=&doi=&projectreference=&title=&author=&leadauthor=&direction=desc&selected_facets=researchtype:%22Evidence%20Synthesis%22) |
| [“street person”](https://www.journalslibrary.nihr.ac.uk/search/#/?search=%22street%20person%22%20&tab=All&page=1&rows=25&orderby=score&publicationrange=publicationdate:%5B*%20TO%20*%5D&chiefinvestigator=&doi=&projectreference=&title=&author=&leadauthor=&direction=desc&selected_facets=researchtype:%22Evidence%20Synthesis%22) |
| [“street people”](https://www.journalslibrary.nihr.ac.uk/search/#/?search=%22street%20people%22%20&tab=All&page=1&rows=25&orderby=score&publicationrange=publicationdate:%5B*%20TO%20*%5D&chiefinvestigator=&doi=&projectreference=&title=&author=&leadauthor=&direction=desc&selected_facets=researchtype:%22Evidence%20Synthesis%22) |
| [shelter](https://www.journalslibrary.nihr.ac.uk/search/#/?search=shelter&tab=All&page=1&rows=25&orderby=score&publicationrange=publicationdate:%5B*%20TO%20*%5D&chiefinvestigator=&doi=&projectreference=&title=&author=&leadauthor=&direction=desc&selected_facets=researchtype:%22Evidence%20Synthesis%22) |
| [vagrant*](https://www.journalslibrary.nihr.ac.uk/search/#/?search=vagrant&tab=All&page=1&rows=25&orderby=score&publicationrange=publicationdate:%5B*%20TO%20*%5D&chiefinvestigator=&doi=&projectreference=&title=&author=&leadauthor=&direction=desc&selected_facets=researchtype:%22Evidence%20Synthesis%22) |
| [“no fixed abode”](file:///C:\Users\Michelle\Documents\Independent%20Training\Harry%20LJMU\journalslibrary.nihr.ac.uk\search\#/?search="no%20fixed%20abode"%20&tab=All&page=1&rows=25&orderby=score&publicationrange=publicationdate:%5B*%20TO%20*%5D&chiefinvestigator=&doi=&projectreference=&title=&author=&leadauthor=&direction=desc&selected_facets=researchtype:"Evidence%20Synthesis" ) |
| [transient](https://www.journalslibrary.nihr.ac.uk/search/#/?search=transient&tab=All&page=1&rows=25&orderby=score&publicationrange=publicationdate:%5B*%20TO%20*%5D&chiefinvestigator=&doi=&projectreference=&title=&author=&leadauthor=&direction=desc&selected_facets=researchtype:%22Evidence%20Synthesis%22) |
| ["unstabl* hous*"](https://www.journalslibrary.nihr.ac.uk/search/#/?search=%22unstabl*%20hous*%22&tab=All&page=1&rows=25&orderby=score&publicationrange=publicationdate:%5B*%20TO%20*%5D&chiefinvestigator=&doi=&projectreference=&title=&author=&leadauthor=&direction=desc&selected_facets=researchtype:%22Evidence%20Synthesis%22) |
| [Hostel*](https://www.journalslibrary.nihr.ac.uk/search/#/?search=hostel*&tab=All&page=1&rows=25&orderby=score&publicationrange=publicationdate:%5B*%20TO%20*%5D&chiefinvestigator=&doi=&projectreference=&title=&author=&leadauthor=&direction=desc&selected_facets=researchtype:%22Evidence%20Synthesis%22) |
| [unshelter*](https://www.journalslibrary.nihr.ac.uk/search/#/?search=unshelter*&tab=All&page=1&rows=25&orderby=score&publicationrange=publicationdate:%5B*%20TO%20*%5D&chiefinvestigator=&doi=&projectreference=&title=&author=&leadauthor=&direction=desc&selected_facets=researchtype:%22Evidence%20Synthesis%22) |
| [roofless*](https://www.journalslibrary.nihr.ac.uk/search/#/?search=roofless*&tab=All&page=1&rows=25&orderby=score&publicationrange=publicationdate:%5B*%20TO%20*%5D&chiefinvestigator=&doi=&projectreference=&title=&author=&leadauthor=&direction=desc&selected_facets=researchtype:%22Evidence%20Synthesis%22) |
| [destitute](https://www.journalslibrary.nihr.ac.uk/search/#/?search=destitute&tab=All&page=1&rows=25&orderby=score&publicationrange=publicationdate:%5B*%20TO%20*%5D&chiefinvestigator=&doi=&projectreference=&title=&author=&leadauthor=&direction=desc&selected_facets=researchtype:%22Evidence%20Synthesis%22) |
| [Runaway*](https://www.journalslibrary.nihr.ac.uk/search/#/?search=Runaway*&tab=All&page=1&rows=25&orderby=score&publicationrange=publicationdate:%5B*%20TO%20*%5D&chiefinvestigator=&doi=&projectreference=&title=&author=&leadauthor=&direction=desc&selected_facets=researchtype:%22Evidence%20Synthesis%22) |
| ["without home*"](https://www.journalslibrary.nihr.ac.uk/search/#/?search=%22without%20home*%22&tab=All&page=1&rows=25&orderby=score&publicationrange=publicationdate:%5B*%20TO%20*%5D&chiefinvestigator=&doi=&projectreference=&title=&author=&leadauthor=&direction=desc&selected_facets=researchtype:%22Evidence%20Synthesis%22) |
| ["without housing"](https://www.journalslibrary.nihr.ac.uk/search/#/?search=%22without%20housing%22&tab=All&page=1&rows=25&orderby=score&publicationrange=publicationdate:%5B*%20TO%20*%5D&chiefinvestigator=&doi=&projectreference=&title=&author=&leadauthor=&direction=desc&selected_facets=researchtype:%22Evidence%20Synthesis%22) |
| ["without house"](https://www.journalslibrary.nihr.ac.uk/search/#/?search=%22without%20house%22&tab=All&page=1&rows=25&orderby=score&publicationrange=publicationdate:%5B*%20TO%20*%5D&chiefinvestigator=&doi=&projectreference=&title=&author=&leadauthor=&direction=desc&selected_facets=researchtype:%22Evidence%20Synthesis%22) |
| ["lack home*"](https://www.journalslibrary.nihr.ac.uk/search/#/?search=%22lack%20home*%22&tab=All&page=1&rows=25&orderby=score&publicationrange=publicationdate:%5B*%20TO%20*%5D&chiefinvestigator=&doi=&projectreference=&title=&author=&leadauthor=&direction=desc&selected_facets=researchtype:%22Evidence%20Synthesis%22) |
| ["lack housing"](https://www.journalslibrary.nihr.ac.uk/search/#/?search=%22lack%20housing%22&tab=All&page=1&rows=25&orderby=score&publicationrange=publicationdate:%5B*%20TO%20*%5D&chiefinvestigator=&doi=&projectreference=&title=&author=&leadauthor=&direction=desc&selected_facets=researchtype:%22Evidence%20Synthesis%22) |
| ["lack house"](https://www.journalslibrary.nihr.ac.uk/search/#/?search=%22lack%20house%22&tab=All&page=1&rows=25&orderby=score&publicationrange=publicationdate:%5B*%20TO%20*%5D&chiefinvestigator=&doi=&projectreference=&title=&author=&leadauthor=&direction=desc&selected_facets=researchtype:%22Evidence%20Synthesis%22) |

**Notes**

NIHR Journals Library does not like long strings of search terms, so each term was searched separately

**JBI Database of Systematic Reviews**

homeless* OR undomiciled OR houseless* OR "rough sleep*" OR shelter OR "street person" OR "street people" OR vagrant* OR "no fixed abode" OR transient OR "unstabl* hous*" OR hostel* OR unshelter* OR roofless* OR destitute OR runaway* OR "without home*" OR "without housing" OR "without house" OR "lack home*" OR "lack housing" OR "lack house"
