# Supplementary material for: What treatment and services are effective for people who are homeless and use drugs? A systematic ‘review of reviews’
Source: PLoS One. 2021 Jul 14;16(7):e0254729. doi: 10.1371/journal.pone.0254729 (PMC8279330; doi:10.1371/journal.pone.0254729)
Supplement: S2 Data — (DOCX) [file pone.0254729.s006.docx]

**S2 Data. JBI critical appraisal checklist for systematic reviews and research syntheses**

JBI Critical Appraisal Checklist for Systematic Reviews and Research Syntheses

Reviewer Date

Author Year Record Number

|  | Yes | No | Unclear | Not applicable |
| --- | --- | --- | --- | --- |
| 1. Is the review question clearly and explicitly stated? | □ | □ | □ | □ |
| 1. Were the inclusion criteria appropriate for the review question? | □ | □ | □ | □ |
| 1. Was the search strategy appropriate? | □ | □ | □ | □ |
| 1. Were the sources and resources used to search for studies adequate? | □ | □ | □ | □ |
| 1. Were the criteria for appraising studies appropriate? | □ | □ | □ | □ |
| 1. Was critical appraisal conducted by two or more reviewers independently? | □ | □ | □ | □ |
| 1. Were there methods to minimize errors in data extraction? | □ | □ | □ | □ |
| 1. Were the methods used to combine studies appropriate? | □ | □ | □ | □ |
| 1. Was the likelihood of publication bias assessed? | □ | □ | □ | □ |
| 1. Were recommendations for policy and/or practice supported by the reported data? | □ | □ | □ | □ |
| 1. Were the specific directives for new research appropriate? | □ | □ | □ | □ |

Overall appraisal: Include □ Exclude □ Seek further info □

Comments (Including reason for exclusion)
