# Supplementary figures and images for: What treatment and services are effective for people who are homeless and use drugs? A systematic ‘review of reviews’
Source: PLoS One. 2021 Jul 14;16(7):e0254729. doi: 10.1371/journal.pone.0254729 (PMC8279330; doi:10.1371/journal.pone.0254729)

**S3 Data. SANRA critical appraisal tool.**


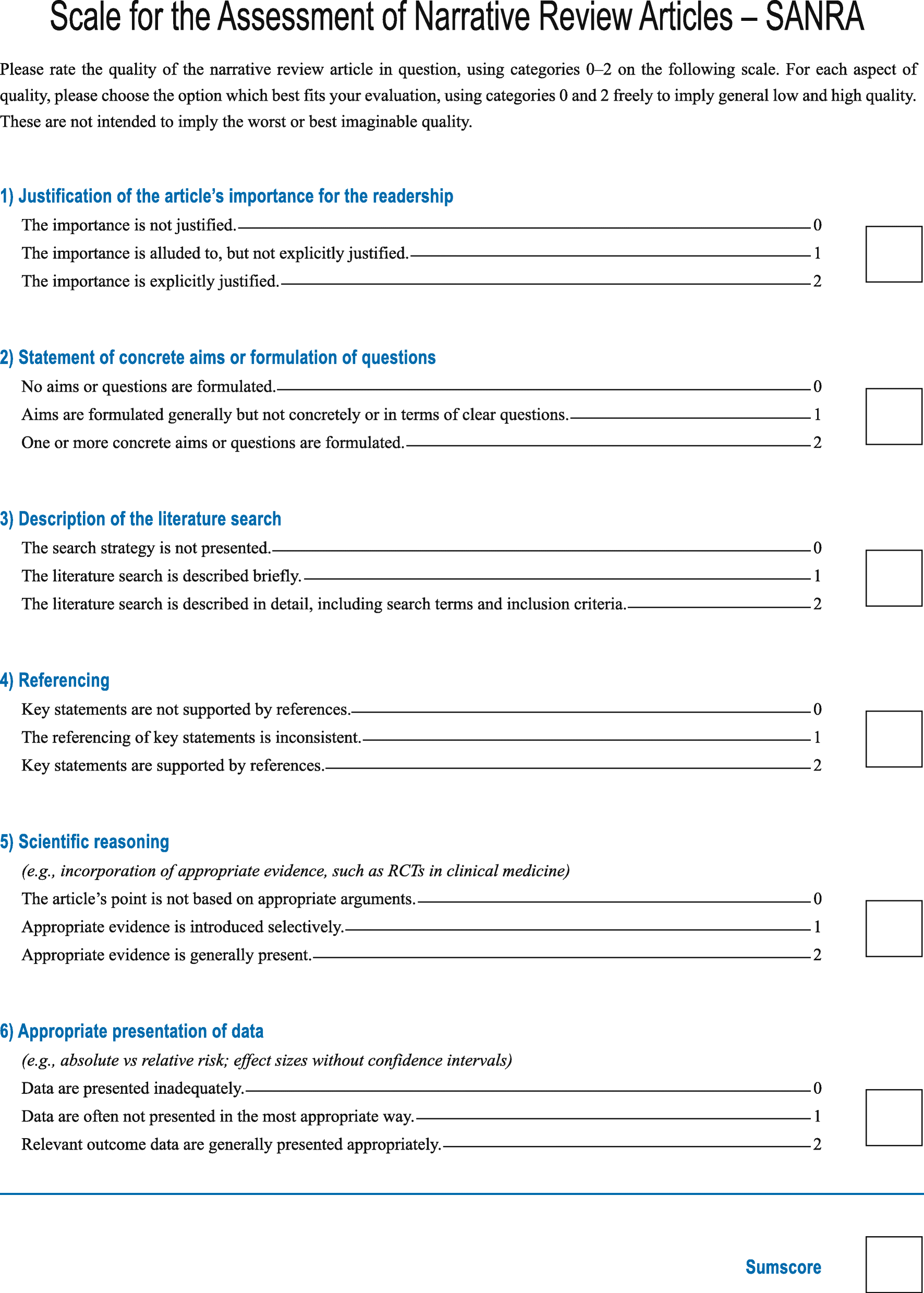

Supplement: S3 Data — (DOCX) [file pone.0254729.s007.docx]
