# Supplementary material for: What treatment and services are effective for people who are homeless and use drugs? A systematic ‘review of reviews’
Source: PLoS One. 2021 Jul 14;16(7):e0254729. doi: 10.1371/journal.pone.0254729 (PMC8279330; doi:10.1371/journal.pone.0254729)
Supplement: S4 Data — (DOCX) [file pone.0254729.s008.docx]

**S4 Data. Abbreviations list**

ACMD - Advisory Council on the Misuse of Drugs

ACT - assertive community treatment

AIDS - acquired immunodeficiency syndrome

BBVs - blood-borne viruses

COH - The Canadian Observatory on Homelessness

COSMHAD - Co-occurring serious mental health problems and alcohol/drug use

CTI – critical time intervention

ED – emergency department

FEANTSA - the **European Federation of National Organisations working with the Homeless** (French: Fédération Européenne d'Associations Nationales Travaillant avec les Sans-Abri)

HAART - highly active antiretroviral therapy

HCV – hepatitis C virus

HF – Housing First

HIV - human immunodeficiency virus

ICM - intensive case management

IPS - Intentional peer support

MET - motivational enhancement therapy

MI – motivational interviewing

NSPs - needle and syringe programmes

OST - opioid substitution therapy

PIE – psychologically informed environments

PRISMA - Preferred Reporting Items of Systematic Reviews and Meta-Analyses

QoL – quality of life

SCFs - supervised consumption facilities

SCM – standard case management

TAU – treatment as usual

TB – tuberculosis

TF – treatment first

THN - take-home naloxone

UK – United Kingdom

USA – United States of America
